# *MMP20*,* KLK4,* and *MMP20/KLK4* double null mice define roles for matrix proteases during dental enamel formation

**DOI:** 10.1002/mgg3.194

**Published:** 2015-12-20

**Authors:** Yuanyuan Hu, Charles E. Smith, Amelia S Richardson, John D. Bartlett, Jan C.C. Hu, James P. Simmer

**Affiliations:** ^1^ Departments of Biologic and Materials SciencesUniversity of Michigan School of Dentistry1210 Eisenhower PlaceAnn ArborMichigan48108; ^2^ Facility for Electron Microscopy ResearchDepartment of Anatomy and Cell Biology and Faculty of DentistryMcGill UniversityMontrealQuebecH3A 2B2Canada; ^3^ Office of ResearchCollege of DentistryOhio State University4139 Postle Hall, 305 W. 12th Ave.ColumbusOhio 43210

**Keywords:** Amelogenesis imperfecta, enamelysin, FAM83H, Kallikrein‐related peptidase 4, matrix metalloproteinase 20

## Abstract

Matrix metalloproteinase 20 (MMP20) and kallikrein‐related peptidase 4 (KLK4) are secreted proteinases that are essential for proper dental enamel formation. We characterized and compared enamel formed in wild‐type, *Mmp20* ^−/−^, *Klk4* ^−/−^, *Mmp20* ^+/−^ *Klk4* ^+/−^, and *Mmp20* ^−/−^ *Klk4* ^−/−^ mice using dissecting and light microscopy, backscattered scanning electron microscopy (bSEM), SEM, microcomputed tomography (*μ*CT), and energy‐dispersive X‐ray analysis (EDX). Following eruption, fractures were observed on *Mmp20* ^−/−^, *Klk4* ^−/−^, *Mmp20* ^+/−^ *Klk4* ^+/−^, and *Mmp20* ^−/−^ *Klk4* ^−/−^ molars. Failure of the enamel in the *Mmp20* ^+/−^ *Klk4* ^+/−^ molars was unexpected and suggested that digenic effects could contribute to the etiology of amelogenesis imperfecta in humans. Micro‐CT analyses of hemimandibles demonstrated significantly reduced high‐density enamel volume in the *Mmp20* ^−/−^ and *Klk4* ^−/−^ mice relative to the wild‐type, which was further reduced in *Mmp20* ^−/−^ *Klk4* ^−/−^ mice. bSEM images of 7‐week *Mmp20* ^−/−^ and *Mmp20* ^−/−^ *Klk4* ^−/−^ mandibular incisors showed rough, pitted enamel surfaces with numerous indentations and protruding nodules. The *Mmp20* ^+/−^ and *Mmp20* ^+/−^ *Klk4* ^+/−^ incisors showed prominent, evenly spaced, horizontal ridges that were more distinct in *Mmp20* ^+/−^ *Klk4* ^+/−^ incisors relative to *Mmp20* ^+/−^ incisors due to the darkening of the valleys between the ridges. In cross sections, the *Mmp20* ^−/−^ and *Mmp20* ^−/−^ *Klk4* ^−/−^ exhibited three distinct layers. The outer layer exhibited a disturbed elemental composition and an irregular enamel surface covered with nodules. The *Mmp20* null enamel was apparently unable to withstand the sheer forces associated with eruption and separated from dentin during development. Cells invaded the cracks and interposed between the dentin and enamel layers. MMP20 and KLK4 serve overlapping and complementary functions to harden enamel by removing protein, but MMP20 potentially serves multiple additional functions necessary for the adherence of enamel to dentin, the release of intercellular protein stores into the enamel matrix, the retreat of ameloblasts to facilitate thickening of the enamel layer, and the timely transition of ameloblasts to maturation.

## Introduction

Two secreted proteinases are essential for dental enamel formation: matrix metalloproteinase 20 (MMP20; OMIM *604629) and kallikrein‐related peptidase 4 (KLK4; OMIM *603767) (Lu et al. 2008). MMP20 is secreted early during the secretory stage, and cleaves or “processes” enamel matrix proteins at a limited number of sites (Ryu et al. 1999). MMP20 cleaves amelogenin (Nagano et al. 2009) and ameloblastin (Iwata et al. 2007; Chun et al. 2010) in vitro at the same sites that must be hydrolyzed in vivo to explain the spectrum of accumulated cleavage products that are found in developing pig enamel (Yamakoshi et al. 2003; Yamakoshi 2011). Uncleaved enamel proteins are prominent in the secretory stage enamel of *Mmp20* null mice (Yamakoshi et al. 2011). MMP20 is expressed by both odontoblasts and ameloblasts at the onset of enamel formation, during formation of the dentino‐enamel junction (DEJ) (Begue‐Kirn et al. 1998), and is critical for proper formation of the interface between dentin and enamel (Beniash et al. 2006). Early during dental biomineralization, a line of hypermineralization along the DEJ can be observed until the overlying enamel reaches a similar density (Hu et al. 2011a). This line is absent during early amelogenesis in *Mmp20* null mice (Hu et al. 2011b), and the enamel layer in these mice fails at the DEJ (Simmer et al. 2012b). *Mmp20* null mice show severe enamel malformations (Caterina et al. 2002; Bartlett et al. 2004, 2011b), with no phenotype evident outside of the dentition. Mutations in human *MMP20* cause a nonsyndromic form of amelogenesis imperfecta (Kim et al. 2005; Papagerakis et al. 2008; Wang et al. 2013; Seymen et al. 2015).

The *Mmp20* gene is found in teleosts (Kawasaki and Suzuki 2011), so its existence preceded the innovation of enamel formation in fish with lungs (Kawasaki and Amemiya 2014). Despite this, MMP20 is clearly specialized for enamel formation. The human expressed sequence tag (EST) database (which does not have a sampling for developing teeth) lists only 4 MMP20 ESTs out of over 3.3 million ESTs characterized for normal tissues, suggesting there is only trace expression of *MMP20* in nondental tissues. *Mmp20* has been independently pseudogenized in many vertebrates that have lost the ability to make teeth or dental enamel during evolution, such as birds (Kawasaki and Suzuki 2011), baleen and sperm whales (Meredith et al. 2011), turtles, pangolins, sloths, and aardvarks (Meredith et al. 2014). The independent degeneration of *Mmp20* in vertebrates that stop making enamel demonstrates a lack of selection pressure for maintaining this gene, except for enamel formation.

Three enamel proteins are secreted along with MMP20: amelogenin, enamelin, and ameloblastin (Fincham et al. 1999). These proline/glutamine‐rich proteins are all members of the secretory calcium‐binding phosphoprotein (SCPP) family (Kawasaki and Weiss 2003), and are found in the coelacanth and lungfish, but not in teleosts (Kawasaki and Amemiya 2014). These three SCPP proteins are specialized for dental enamel formation and their appearance during evolution is associated with the development of a specialized mineralization front apparatus along the distal membrane of ameloblasts that generates, extends and orients numerous thin mineral ribbons beneath each cell. This process is the defining feature of true enamel and is remarkably similar in lungfish and in mammals (Satchell et al. 2000; Ronnholm 1962a). Amelogenin, enamelin, and ameloblastin are secreted and partially reabsorbed by ameloblasts at the mineral front as the ribbons elongate. The target specificity of MMP20 is an important factor in determining which protein domains accumulate and which are reabsorbed.

The onset of KLK4 expression is later than MMP20 and occurs during the transition by ameloblasts into the maturation stage (Simmer et al. 2011b; Hu et al. 2000, 2002). Unlike *Mmp20*,* Klk4* is not expressed by odontoblasts (Simmer et al. 2011a). KLK4 cleaves enamel proteins at more sites than MMP20, and serves a more degradative (rather than processing) function. KLK4 cleaves amelogenin at multiple sites in vitro (Ryu et al. 2002), and its cleavage pattern complements that of MMP20, so that the accumulated matrix proteins that have been processed by MMP20 undergo subsequent degradation into smaller polypeptides. Degradation by KLK4 facilitates diffusion of these fragments back to the enamel surface where they are reabsorbed by maturation stage ameloblasts. Enamelin is secreted as a 186‐kDa protein (Hu et al. 1997a), but only a 32‐kDa enamelin cleavage product accumulates to abundance during the secretory stage (Tanabe et al. 1990a). This highly glycosylated peptide (Yamakoshi 1995) resists further degradation by MMP20. In contrast, KLK4 hydrolyzes it into multiple products (Yamakoshi et al. 2006). Although MMP20 can be activated by the KLK4 zymogen in vitro (Ryu et al. 2002), *Klk4* is expressed normally in the *Mmp20* null background and appears as an active band on zymograms of enamel extracts (Yamakoshi et al. 2011). As is the case for MMP20, enamel malformations are evident in *Klk4* null mice, but the phenotype is very different than in the *Mmp20* nulls (Simmer et al. 2009b; Smith et al. 2011b). *Klk4* null mouse enamel is normal in thickness and contour, and the enamel rods show the same decussation pattern as wild‐type teeth, but the enamel is hypomineralized and contains residual enamel proteins. Despite the deficiency in removing enamel proteins from the matrix, mineralization of the maturation stage enamel matrix proceeds normally until the mineral level reaches ~80% of normal (Smith et al. 2011c), and then stalls, apparently because residual protein occupies the space between the crystals and blocks completion of mineralization where the crystals grow into contact and interlock.

*Klk4* is the most recently evolved of the kallikrein‐related peptidase genes (Kawasaki et al. 2014), a family that in humans includes 15 members, all clustered on the long arm of chromosome 19. The *KLK* family arose from a trypsin‐like gene and is largely a mammalian innovation. *Klk4* was generated from a duplication of *Klk5* and is found only in boreoeutherian mammals, where it appears to have enhanced enamel maturation, enabling increased enamel thickness or earlier tooth eruption without reducing enamel hardness (Kawasaki et al. 2014). In this study we further characterize *Mmp20‐* and *Klk4*‐deficient mice and *Mmp20Klk4* (MK) double null mice and discuss the findings with respect to current theories of enamel biomineralization.

## Materials and Methods

### Ethical compliance

All procedures involving animals were reviewed and approved by the UACUC committee at the University of Michigan.

### Generation of *MK* double knockout mice

*Mmp20* null mice in the C57BL/6 background (Caterina et al. 2002) were mated with *Klk4* null mice, also in the C57BL/6 background (Simmer et al. 2009b) to generate double heterozygous offspring (*Mmp20* ^+/−^ *Klk4* ^+/−^ or *M* ^+/−^ *K* ^+/−^), which were mated to obtain *Mmp20Klk4* (*MK*) double null mice (*Mmp20* ^−/−^ *Klk4* ^−/−^ or *M* ^−/−^ *K* ^−/−^), which were interbred to maintain that genotype. Genotyping was accomplished using five pairs of PCR primers that together produce a unique pattern for each genotype (Fig. S1).

### Dissecting microscopy

Day 14, Day 17, and 9‐week‐old (adult) mice were anesthetized with isoflurane, sacrificed by head dislocation, and fixed by immersion in 4% paraformaldehyde (PFA). The mandibles were removed and dissected free of soft tissues. The teeth were cleaned with nonwoven gauze, displayed on the Nikon SMZ1000 dissection microscope and photographed using a Nikon digital camera DXM1200.

### Backscattered scanning electron microscopy

The backscattered scanning electron microscopy (bSEM) procedures were described previously (Smith et al. 2011c). For enamel thickness measurements, soft tissues were removed from left and right hemimandibles of wild‐type, *Klk4* ^−/−^, *Mmp20* ^−/−^ and *Mmp20* ^−/−^ *Klk4* ^−/−^ (*MK* double null) mice at 7 weeks, sectioned at the level of the labial alveolar ridge (Fig. S2), and imaged by bSEM. Incisor and molar imaging was performed at 7 weeks on wild‐type, *Klk4* ^+/−^, *Klk4* ^−/−^, *Mmp20* ^+/−^, *Mmp20* ^−/−^, and *Mmp20* ^−/−^ *Klk4* ^−/−^ (*MK* double null) mice. For incisor imaging, the bony caps and soft tissue covering the mandibular incisors were carefully removed, and examined at ×50 magnification in a Hitachi S‐3000N variable pressure scanning electron microscope using the backscatter mode at 25 kV and 20 pascal pressure. For molar imaging, soft tissues were dissected away, the crowns wiped clean and air‐dried and then imaged in backscatter mode at ×40 magnification using 15–30 kV and 20 pascal pressure.

### Scanning electron microscopy

Scanning electron microscopy (SEM) evaluation was performed at the University of Michigan Microscopy and Image‐analysis Laboratory (Ann Arbor, MI). Ethanol dehydrated, air‐dried hemi‐mandibles and mandibular incisors from Day 17 wild‐type, *Klk4* ^−/−^, *Mmp20* ^−/−^, and *Mmp20* ^−/−^ *Klk4* ^−/−^ mice were mounted on metallic stubs using conductive carbon cement, degassed in a vacuum desiccator overnight, and sputter‐coated with an Au‐Pd film to increase conductivity. The samples were imaged using an Amray EF 1910 Scanning Electron Microscope operating at an accelerating voltage of 3–5 kV.

### Wavelength‐dispersive X‐ray spectroscopy

Hemimandibles from wild‐type (WT), *Klk4* null, *Mmp20* Null, and *MK* double null mice were collected at 9 weeks, freeze‐dried, cleared of all soft tissue, and embedded in Castolite AC (Eager Polymers, Chicago, IL). The embedded hemimandibles/incisors were cut transversely at the level of the labial alveolar crest and embedded again with Castolite AC in 25 mm SeriForm molds (Struers Inc, Westlake, OH). The incisor cross sections were successively polished with 120, 180, 400, and 800 grit waterproof silicon carbide papers, followed by diamond polishing overnight. The polished incisor cross sections were imaged and analyzed using a Cameca SX100 Electron Microprobe Analyzer at University of Michigan Electron Microbeam Analysis Laboratory (EMAL). The spot beam used a 15 kV accelerating voltage and 2 nA beam current. The standard (Wilberforce, Ontario CA) for Ca and P was natural apatite, for Na was natural albite, for Mg was natural enstatite, for Cl was natural scapolite, and for K was natural adularia. The Ca, P, Mg, Na, K, and Cl atomic percentages were analyzed along a line from near the dentin surface outward to near the enamel surface. Control points were also sampled from inner dentin, middle dentin, outer dentin, and alveolar bone. Wavelength‐dispersive X‐ray spectroscopy (WDX) data were collected from 20 points for each mouse genotype. Statistical analyses used the Microsoft Excel *t*‐test.

### Histological staining and analyses

Wild‐type, *Mmp20* ^−/−^, *Klk4* ^−/−^, *Mmp20* ^−/−^ *Klk4* ^−/−^, *Mmp20* ^+/−^, *Klk4* ^+/−^, and *Mmp20* ^+/−^ *Klk4* ^+/−^ mice at 7‐weeks were deeply anesthetized with isoflurane, fixed by cardiac perfusion with 2.5% glutaraldehyde in 0.1 mol/L sodium cacodylate buffer (pH 7.2–7.4) containing 0.05% calcium chloride, postfixed for 2 h at 4°C, and rinsed 3× for 15 min each with 0.1 mol/L sodium cacodylate buffer. The samples were decalcified at 4°C by immersion in 1 L of 4.13% disodium ethylenediaminetetraacetic acid (EDTA, pH 7.3) with agitation. The EDTA solution was changed every other day for 30 days. The samples were washed in PBS at 4°C 4–5 times every 0.5–1 h, washed overnight, postfixed for 60 min in 1% osmium tetroxide in 1.5% potassium ferrocyanide, and dehydrated using an acetone gradient, embedded in Epon812 substitute, and semi thin‐sectioned and stained with 0.1% toluidine blue as described elsewhere (Smith et al. 2011a). At least three maxillary and three mandibular incisors were processed for longitudinal sectioning, and three mandibular incisors were processed for cross sectioning at 1 mm increments at the approximate locations shown in Figure S2C.

## Results

### Gross morphology and attrition

**Figure 1:**
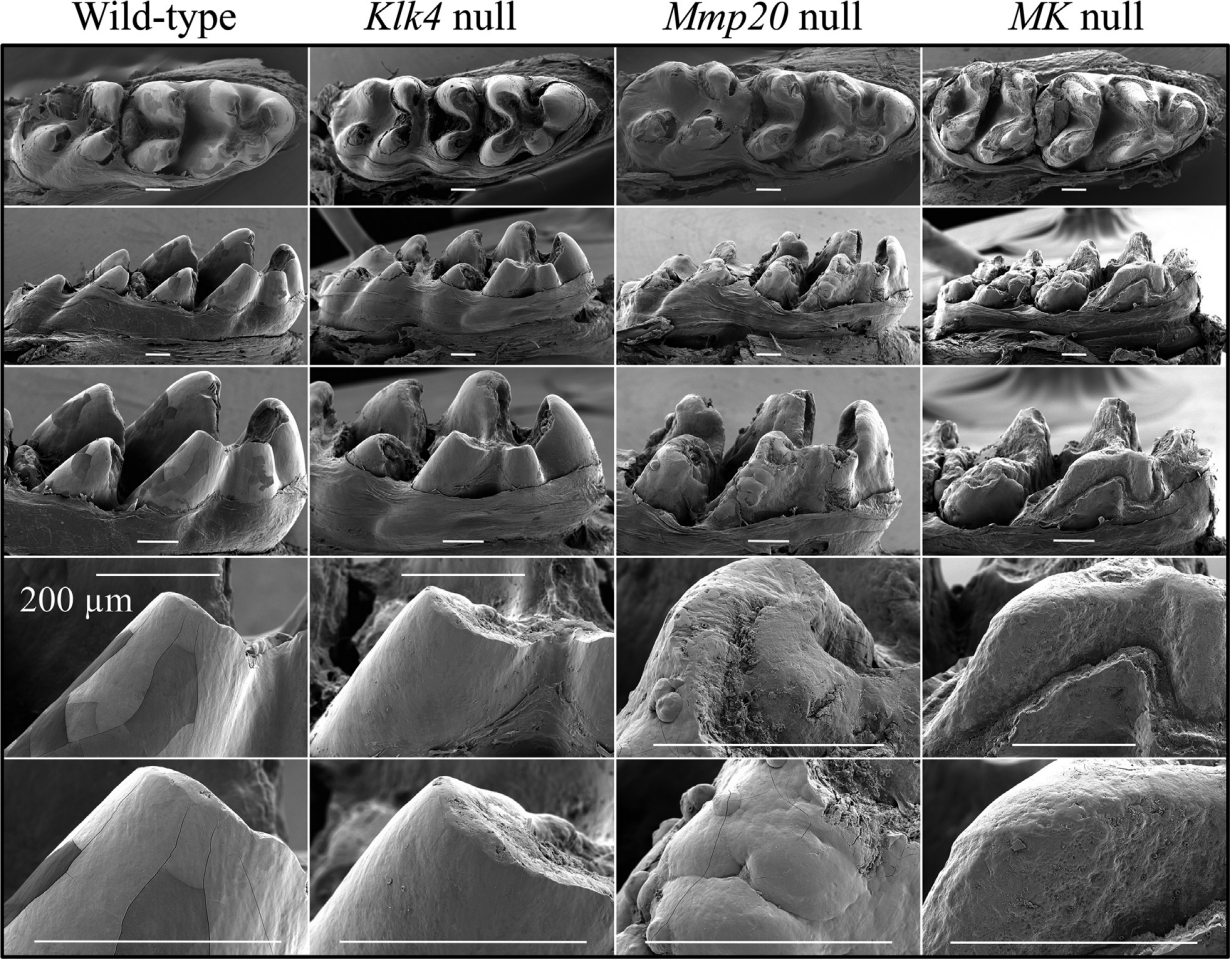
Scanning electron microscopy of Day 17 mandibular molar crowns. Descending from top row: occlusal view, lingual view (M2 on left; M1 on right), lingual view of M1, and progressively higher magnification views of the distal‐lingual cusp. These images were take only a day or two following eruption. The overall enamel form of wild‐type and *Klk4* null molars is the same. The enamel crust of *Mmp20* null molars is irregular and shows mineral nodules of various sizes protruding from the surface. The *Mmp20/Klk4* double null molars appeared to be very similar to *Mmp20* ^*−/−*^ molars, but had erupted slightly earlier and already showed significant attrition. Bars = 200 *μ*m.

The gross morphologies of the mouse molar and incisors before and after eruption were assessed under a dissecting microscope, by scanning electron microscopy (SEM) and by bSEM. Mandibular molars and incisors were examined at Day 14 (following the removal of soft tissues) prior to eruption of the first molars (Fig. S3) and at Day 17, shortly following eruption of the first molars (Fig. 1, Fig. S4). This allowed us to first evaluate the molar crowns before they had erupted and potentially been altered in the oral cavity, and to determine how quickly they failed following eruption. Prior to and immediately after eruption, the molar crowns and incisors of *Klk4* null mice looked similar to those of the wild‐type (WT) mice. The cusps showed normal contour and the enamel surfaces were both smooth and reflective. In contrast, *Mmp20* null and *MK* double null (*Mmp20* ^*−/−*^ *Klk4* ^−/−^) molars had thinner cusps with rough, dull surfaces. Immediately following eruption both *Mmp20* and *MK* double null first molars were already severely damaged, especially on the cusps (working surfaces) (Fig. 1). Enamel that had not worn away was irregular, with dome‐like nodules protruding from the surface. By week 7 (5 weeks after the eruption of the mandibular first molars), severe enamel attrition was observed on all molars and incisors in *Klk4* null, *Mmp20* null, and *MK* double null mice (Fig. S5). This was true even though all mice were maintained on soft chow.

**Figure 2:**
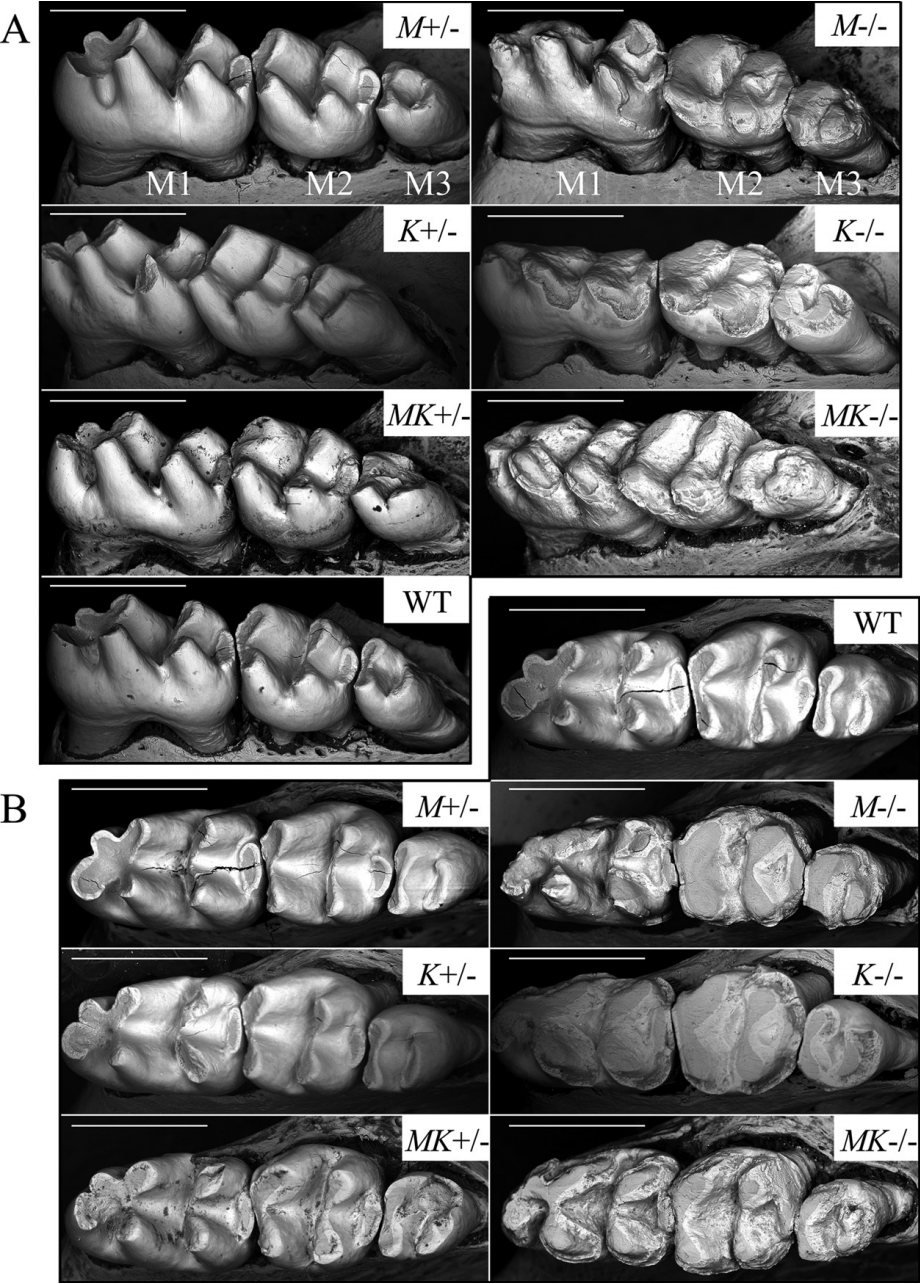
Backscatter scanning electron microscopy of 9‐week mandibular molar crowns. (A) Buccal‐occlusal views (M1 first molar; M2 second molar; M3 third molar). (B) Occlusal views. These molars have been in occlusion for as much as 48 days (first molars) while the animals were maintained on a soft diet. The *Mmp20* null (*M*−/−), *Klk4* null (*K*−/−), and *MK* double null (*MK*−/−), molars show severe enamel abrasion that exposes the underlying dentin. The *Mmp20* (*M*+/−) and *Klk4* (*K*+/−) heterozygous molars are similar to the wild‐type and show no signs of attrition. The *MK* double heterozygous (*MK*+/−) molars sometimes showed significant attrition (Note the distal buccal surface of the third molar). Cracks are not part of the phenotype, but are unavoidable artifacts from freeze drying. Bars = 1 mm.

Mandibular molars (9 week) from wild‐type, *Mmp20* ^+/−^, *Mmp20* ^*+/−*^ *Klk4* ^+/−^, *Mmp20* ^−/−^, *Klk4* ^−/−^, and *Mmp20* ^*−/−*^ *Klk4* ^−/−^ mice were imaged using backscatter scanning electron microscopy (Fig. 2). Wild‐type molars showed a smooth enamel layer covering the entire crown except in the enamel free zones at the cusp tips. *Mmp20* and *Klk4* heterozygous mouse molars were indistinguishable from the wild‐type. Notably, *MK* double heterozygous mouse molars often appeared normal in form, but showed increased surface roughness and a susceptibility to attrition: the enamel fractured off the double heterozygous crowns in some places. This unexpected finding suggested that an enamel phenotype can result from digenic inheritance (Schaffer 2013), which has not yet been demonstrated in human cases of amelogenesis imperfecta. Both *Mmp20* and *Klk4* single null mice showed severe occlusal attrition, which appeared to be even worse in *MK* double null mice.

### Enamel thickness

**Figure 3:**
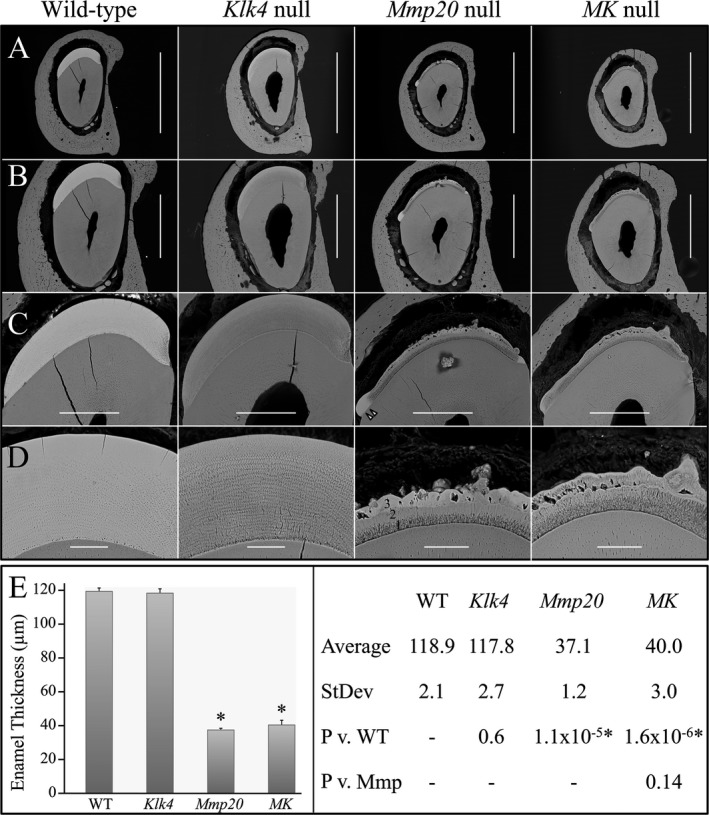
Backscattered scanning electron microscopy (bSEM) analysis and enamel thickness of mandibular incisor cross sections at level 6 from 7‐week‐old wild‐type, *Klk4* null, *Mmp20* null, and *MK* double null mice. (A) Scale Bar = 1 mm. (B) Scale Bar = 500 *μ*m. (C) Scale Bar = 200 *μ*m. (D) Scale Bar = 50 *μ*m. The enamel layers of wild‐type and *Klk4* null mice are virtually identical in both their thickness and rod decussation patterns. The *Klk4* null enamel is hypomineralized relative to the wild‐type. The hypomineralization is more severe with depth, and most severe at the junction of the initial enamel and rod enamel, just above the dentino‐enamel junction (DEJ). The enamel layers of *Mmp20* and *MK* double null mice both show a dark, poorly mineralized band at the DEJ overlaid by three morphologically distinct mineralized layers. (E) Statistical analyses of enamel thickness. The enamel thickness of *Klk4* null mice is the same as wild‐type mice, whereas *Mmp20* null and *MK* double null mice both have rough enamel layers that are only a third as thick as the wild‐type on average. Key: Averages are in *μ*m; *P* = *P* value.

Enamel thickness was measured by bSEM of polished cross sections 8 mm from the basal end of 7‐week mandibular incisor in wild‐type, *Mmp20*,* Klk4*, and *MK* double null mice (Fig. 3). Sections at this level (where the enamel of the continuously erupting incisor has matured and erupted to the level of the alveolar crest) sample the middle to late maturation stage well after the enamel layer has reached its final thickness, but before it has entered the oral cavity and undergone attrition. The bSEM images of polished sections were used to assess enamel thickness, rod decussation pattern and relative enamel mineralization levels in the various genetic backgrounds. The enamel layers in the wild‐type and *Klk4* null mice were both ~115‐*μ*m thick and showed normally decussating enamel rods. *Klk4* null enamel was less mineralized than the wild‐type enamel throughout, but was also progressively less mineralized with depth, and least mineralized just above the DEJ. The *Mmp20* and *MK* double null enamel layers were similar to each other, but fundamentally different from those of the wild‐type and *Klk4* null mice. Thickness of the enamel layer in *Mmp20* and *MK* double null mandibular incisors was variable due to the presence of surface irregularities and nodules. Enamel layer thickness in these mice was, on average, only about one‐third that of wild‐type enamel, and was comprised of three distinct mineral layers (Fig. 3D). Superficial to a line of severe hypomineralization at the DEJ laid a zone, 10–15 *μ*m in thickness, of poorly mineralized enamel, well below the density of dentin. This layer was characterized by irregular light and dark bands oriented roughly perpendicular to the DEJ. Superficial to this layer was a middle zone that was about the same thickness (10–15 *μ*m) as the deeper layer, but more homogeneous and about the same density as dentin. This layer was more distinct in *Mmp20* nulls and more highly mineralized than the same layer in *MK* double nulls. In the *MK* double nulls there were regions where the second layer appeared to be morphologically similar to the first layer in that the degree of mineralization was very low and irregular. Despite this, the boundary between the first and second layers was apparent throughout. The third, most superficial layer, was highly mineralized, almost as dense as wild‐type enamel in some areas, and interrupted by unmineralized vacancies apparently caused by entrapped cells. This layer varied greatly in thickness, with mineral nodules projecting from its surface in some places. This third layer was less highly mineralized in *MK* double null mice relative to *Mmp20* single null mice, except for the most superficial 2–3 *μ*m, which was highly mineralized in both cases. The distal enamel margin, near where the enamel layer ends and the root analog begins, was expanded and more highly mineralized in *Mmp20* null mice relative to MK double null mice. This analysis demonstrates that there are fundamental problems with the deposition of enamel in *Mmp20* and *MK* double null mice. Layers 1 and 2 appeared to correspond to inner and outer enamel, respectively, which in wild‐type mice differ primarily in the orientations of their enamel rods, although the rods lack definition in *Mmp20* and *MK* double null enamel. The *Klk4* null enamel layer showed a normal rod pattern and final thickness, but diminished mineralization.

### Incisor enamel surface features

**Figure 4:**
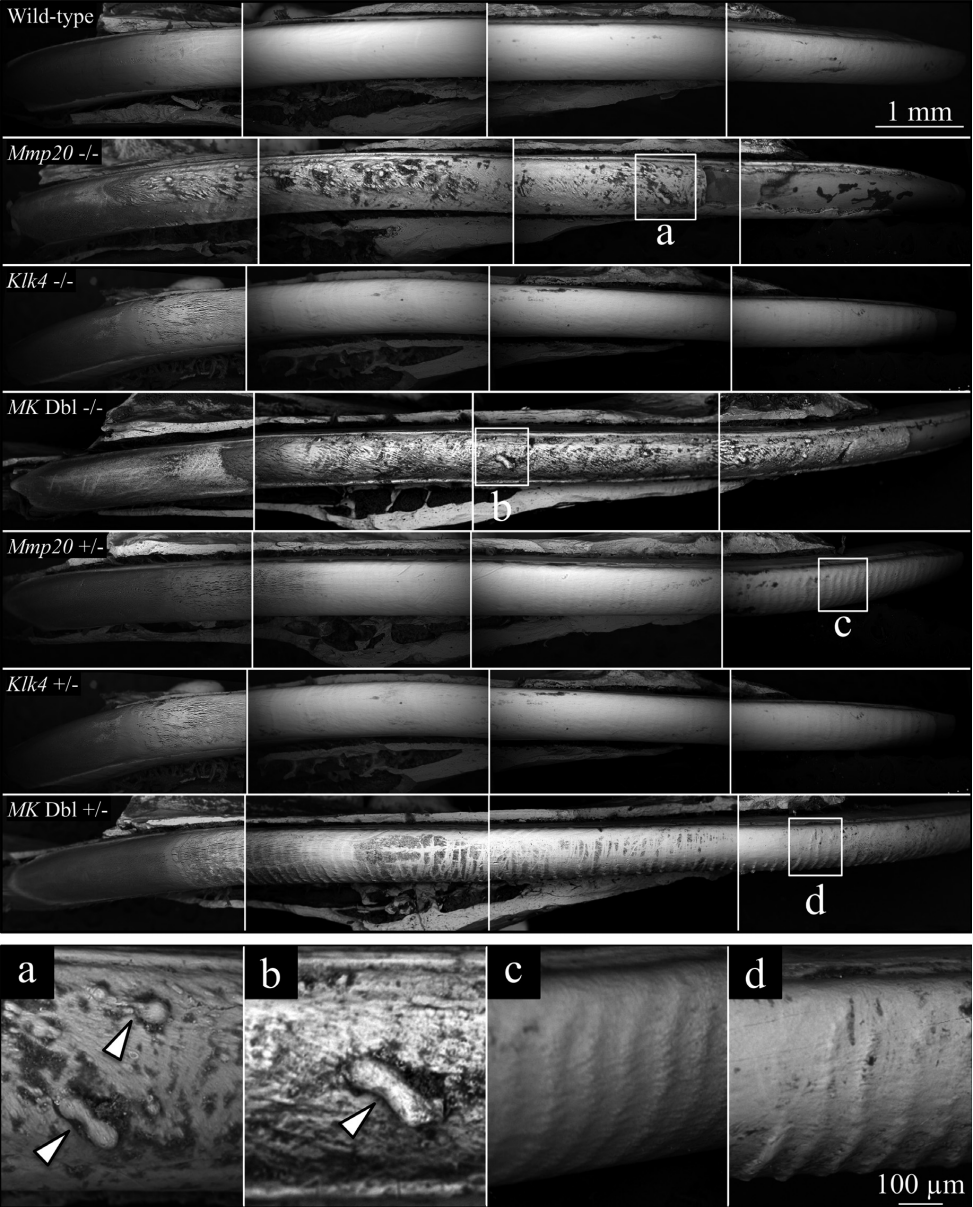
Backscatter scanning electron microscopy of 7‐week mandibular incisors denuded of overlying tissues. The labial surfaces are shown, from the basal (left) to erupted (right) ends of the incisors. Dark areas contain less surface mineral than white areas. The labial surfaces of incisors from wild‐type, *Klk4* null and *Klk4* heterozygous mice were relatively smooth‐surfaced and evenly mineralized. In contrast, the labial incisor surfaces from heterozygous *Mmp20* null and *MK* double null mice had rough, pitted surfaces with indentations and protruding nodules (arrowheads in a and b, respectively). *Mmp20* heterozygous mice and *MK* double heterozygous mice both showed prominent horizontal ridges evenly spaced ~90 *μ*m apart (detailed in c and d, respectively). The ridges in *MK* double heterozygous mice were more distinct in the central regions of the incisor relative to *Mmp20* single‐heterozygous mice due to the darkening of the valleys between the ridges.

Rodent incisors are noteworthy because all of the stages of amelogenesis are linearly arrayed in the continuously growing incisors (Smith and Nanci 1989). In addition, the incisors are continuously erupting, which applies shear forces to the enamel organ during amelogenesis. Mandibular incisors (7 week) from wild‐type, *Mmp20* ^+/−^, *Klk4* ^+/−^, *Mmp20* ^*+/−*^ *Klk4* ^+/−^, *Mmp20* ^−/−^, *Klk4* ^−/−^, and *Mmp20* ^*−/−*^ *Klk4* ^−/−^ mice were carefully dissected and their exposed enamel surfaces were imaged by bSEM (Fig. 4). The incisors of *Klk4* ^+/−^ mice were indistinguishable from wild‐type incisors. An interesting and unexpected finding from the bSEM images was that *Mmp20* ^*+/−*^ *Klk4* ^+/−^ and *Mmp20* ^+/−^ incisor enamel showed distinct stripes, or evenly spaced horizontal ridges about 90 *μ*m apart. These ridges were obvious along the entire length of maturation stage enamel in *Mmp20* ^*+/−*^ *Klk4* ^+/−^ incisors, with the valleys between the ridges being less highly mineralized than the ridges themselves. The striped pattern appeared more C‐shaped basally and S‐shaped incisally. The ridges were generally less apparent in *Mmp20* ^+/−^ incisor enamel, but were detectable and increasingly prominent incisally. Close inspection of the other incisors showed traces of similar bands in *Klk4* ^+/−^, *Klk4* ^−/−^, and wild‐type mouse incisors. The spacing of these lines is not consistent with patterns of ameloblast modulation and may be caused by stresses associated with eruption. The intensity of the superficial banding was greatest in *Mmp20* ^*+/−*^ *Klk4* ^+/−^ incisors and decreased in the sequence *Mmp20* ^+/−^>*Klk4* ^−/−^>*Klk4* ^+/−^> wild‐type. Band intensity in double heterozygous (*Mmp20* ^*+/−*^ *Klk4* ^+/−^) mice was more distinct than in either of the single‐heterozygous mice, demonstrating a digenic contribution by the two enamel matrix protease genes for an enamel phenotype. The banding was presumably not observed in *Mmp20* ^*−/−*^ *Klk4* ^−/−^ and *Mmp20* ^−/−^ nulls because the severity of the enamel defects obscured the banding patterns, or the effects of the eruption forces were manifested differently, as in the shearing off of the enamel layer. The enamel layers in *Mmp20* ^*−/−*^ *Klk4* ^−/−^ and *Mmp20* ^−/−^ incisors were thin and frail, with a lumpy surface. The enamel delaminated near where the incisor erupted. *Mmp20* ^*−/−*^ *Klk4* ^−/−^ and *Mmp20* ^−/−^ mice both displayed rough enamel surfaces that were highly variable in their degree of mineralization. The coarse enamel had nodules protruding from the surface. These nodules were typically irregular, but occasionally were spherical or tubular in form.

### Volume of high‐density mineral

**Figure 5:**
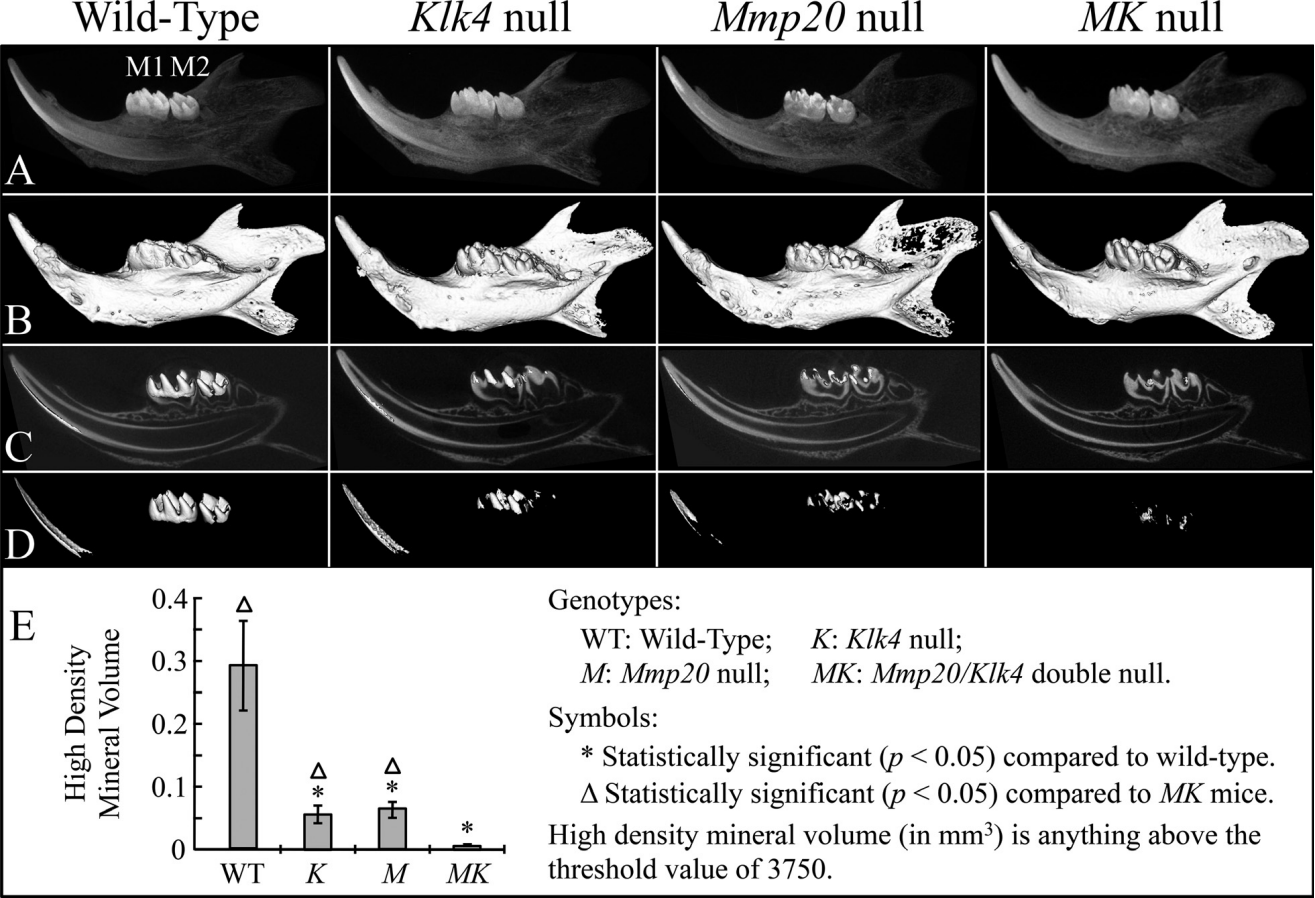
Microcomputed tomography (*μ* CT) of day 14 mouse hemimandibles. (A) Radiographs of the hemimandibles (M1 first molar; M2 second molar).(B) 3D *μ* CT of the hemimandibles. (C) 2D *μ* CT of the hemimandibles with the tooth crowns replaced with 3D images. (D) 3D images of mineral above the threshold value of 3750. Note that none of the incisor enamel and only small areas of the molar enamel reaches the threshold level. (E) Plot of high‐density volume (mm^3^) showing that both *Mmp20* and *Klk4* single null mice have significantly reduced high‐density enamel relative to the wild‐type, but significantly more than *MK* double null mice.

Hemimandibles from the various mouse genetic backgrounds at day 14 were imaged by radiography and 3D and 2D microcomputed tomography (*μ*CT) (Fig. 5). The threshold value for imaging was raised until enamel was the only mineral displayed in the wild‐type. The suprathreshold incisor enamel on the wild‐type image started close to the position of section level 7, indicating that the threshold value of 3750 only permitted imaging of mineral as dense as that of mid to late maturation stage enamel. Both *Klk4* and *Mmp20* single null mice showed significantly reduced volume of high‐density mineral relative to the wild‐type, but significantly more high‐density mineral volume than in *Mmp20* ^*−/−*^ *Klk4* ^−/−^ mice. Almost no enamel in *Mmp20* ^*−/−*^ *Klk4* ^−/−^ mice exceeded the high‐density threshold. Therefore, the volume of high‐density mineral showed a significant digenic phenotype in the null conditions.

### Energy‐dispersive X‐ray analysis

**Figure 6:**
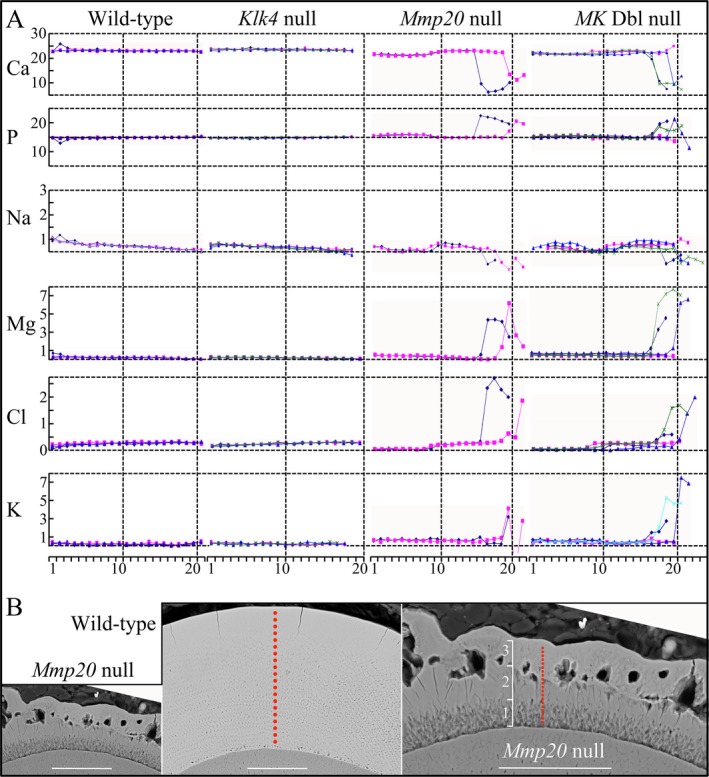
Enamel elemental compositions. (A) Plots of Ca, P, Na, Mg, Cl, and K atomic percentages against location in the enamel from the dentino‐enamel junction (DEJ) to the surface for wild‐type, *Klk4* null, *Mmp20* null, and *MK* double null mice. The multiple colored lines indicate samples taken from different mice. Note that none of the atomic percentages change in wild‐type or *Klk4* null enamel. Elemental compositions in the enamel near the DEJ are virtually the same in all genotypes. The elemental compositions in the *Mmp20* and *MK* double null incisors differ sharply near the enamel surface, most notably showing lower Ca and higher P. (B) backscattered scanning electron microscopy (bSEMs) of mandibular incisors cross‐sectioned at level 8. Red dots map the locations of samples taken for elemental analysis. Left and middle panels show *Mmp20* null and wild‐type enamel sections at the same magnification (Bar = 50 *μ*m); right panel is higher magnification of *Mmp20* null enamel to distinguish the three enamel layers and the sampling sites. Bar = 94 *μ*m.

Elemental compositions (Ca, P, Na, Mg, Cl, and K) of the enamel from the DEJ to the surface were characterized for wild‐type, *Mmp20* ^*−/−*^, *Klk4* ^−/−^, and *Mmp20* ^*−/−*^ *Klk4* ^−/−^ mice (Fig. 6). The elemental compositions for wild‐type and *Klk4* null mice were indistinguishable at all points analyzed. The first two mineral layers of *Mmp20* ^*−/−*^ *Klk4* ^−/−^ and *Mmp20* ^*−/−*^ mouse enamel showed the same elemental compositions as did the wild‐type; however, the elemental compositions of the third mineral layer in these null mice was generally lower in Ca, and higher in P, Na, Mg, Cl, and K than wild‐type enamel. The superficial layers in *Mmp20* ^*−/−*^ and *Mmp20* ^*−/−*^ *Klk4* ^−/−^ mice were fundamentally different in their elemental compositions than wild‐type enamel and appeared to be pathological in origin (not formed by the deposition of mineral ribbons at the mineralization front).

### Histology

To better understand how the pathological enamel formed in the various genotypes, extensive histological characterizations were conducted. Incisor cross sections were characterized at 1 mm increments along 7‐week‐old mandibular incisors from wild‐type (Fig. S6), *Klk4* ^−/−^ (Fig. S7), *Mmp20* ^−/−^ (Fig. S8), *Mmp20* ^−/−^ *Klk4* ^−/−^ (Fig. S9), *Klk4* ^+/−^ (Fig. S10), *Mmp20* ^+/−^ (Fig. S11), and *Mmp20* ^+/−^ *Klk4* ^+/−^ (Fig. S12) mice. In addition, longitudinal sections of 7‐week‐old maxillary and mandibular incisors were characterized from wild‐type (Fig. S13, S14), *Klk4* ^−/−^ (Fig. S15, S16), *Mmp20* ^−/−^ (Fig. S17, S18), *Mmp20* ^−/−^ *Klk4* ^−/−^ (Fig. S19, S20), *Klk4* ^+/−^ (Fig. S21, S22), *Mmp20* ^+/−^ (Fig. S23, S24), *Mmp20* ^+/−^ *Klk4* ^+/−^ (Fig. S25, S26) mice.

The positions along the mandibular incisors of the incisor cross sections are illustrated in Figs S2c. For each genotype, nine incisor cross sections were characterized. Section 1 localized to a position 1 mm from the basal end of the incisor and shows the forming incisor near the onset of dentin and enamel formation. Section 2 shows midsecretory stage. Section 3 shows late secretory stage, whereas sections 4–9 are all at progressively later stages of enamel maturation. Mandibular incisor enamel at its greatest thickness is about 115‐*μ*m thick. In decalcified incisors of wild‐type mice, the enamel space appears to be cleared of protein by level 6 or 7 (Fig. S6). Once the enamel proteins have been largely removed by ameloblasts, the enamel space often gets distorted during histological processing and is not always a good indicator of enamel thickness in demineralized samples. In *Klk4* ^−/−^ mice, enamel formation proceeds normally except that the enamel matrix proteins are not efficiently removed, especially from the deeper enamel, and stained matrix is readily observed even near the point where the incisor erupts out of the gingiva and into the oral cavity (level 9) (Fig. S7).

**Figure 7:**
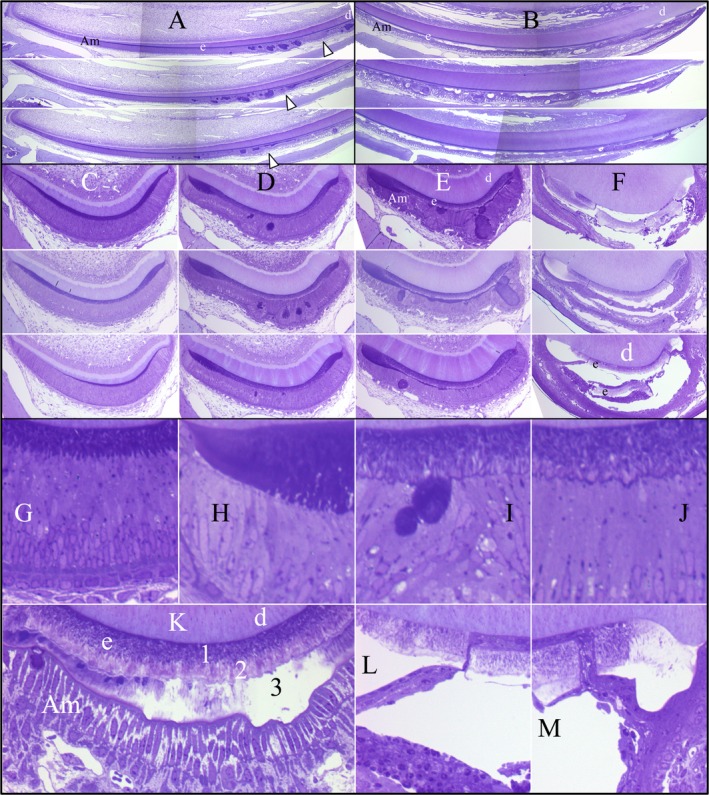
Histology 7‐week *Mmp20* ^*−/−*^ mandibular incisor. (A) Basal ends of mandibular incisor longitudinal sections from three different *Mmp20* ^*−/−*^ mice. Arrowheads mark the beginning and end of the postsecretory transition. (B) Incisal ends of mandibular incisor longitudinal sections from the 3 *Mmp20* ^*−/−*^ incisors shown in A. (C–F) Mandibular incisor cross sections taken from levels 2–4 (C–E) and level 9 (F) from different *Mmp20* ^*−/−*^ mice. (G) Level 2 detail showing secretory stage ameloblasts with Tomes' processes and thin layer of inner enamel. (H) Level 3 detail showing a thickened extracellular matrix near the cervical margin. (I–J) Level 3 details showing secretory ameloblasts atop a thin enamel layer containing both inner and outer enamel layers, and a highly stained line over the dentin surface. Some deeply stained matrix appears to have flowed out of the enamel and bulged into the epithelia on the enamel surface. (K) Level 7 detail showing maturation ameloblasts hardening the third mineral layer atop of the outer enamel. (L–M) Level 9 details showing a segment of enamel has broken off of the dentino‐enamel junction (DEJ) of the continuously erupting incisor and a cell layer that has moved in from the surface. Am, ameloblasts; d, dentin; e, enamel.

MMP20 is active at the onset of enamel formation and its absence has a greater impact on the enamel phenotype than does the absence of KLK4, which is not expressed until the enamel layer has reached its final dimensions. The histological study of the *Mmp20* ^−/−^ mandibular molar histology is summarized in Fig. 7. In *Mmp20* ^−/−^ mice, only a thin layer of enamel matrix is initially deposited, which stains deeply in section 2 (Fig. S8). Already at section level 2, the area occupied by the enamel extracellular matrix is significantly less than in the WT and *Klk4* null mice. The thinness of the enamel layer by itself could cause subsequent developmental problems, as the sheet of ameloblasts covering it must rest on an enamel surface that is smaller than normal. At section level 3, a thin layer of outer enamel that does not stain as intensely as the inner enamel below it forms a second distinct layer over the first. In addition, deeply stained matrix is observed within bulges in the ameloblast layer. The cross sections give the distinct impression that the ameloblast layer has buckled and that the gel‐like extracellular matrix has flowed toward the cervical margins and into wrinkles or blisters in the sheet of ameloblasts, disrupting the linearity of the ameloblast cell layer. The impression of bulk matrix flow is enhanced by the infrequent observation of a channel connecting the enamel matrix with ectopic matrix entirely lined by ameloblasts (Bartlett et al. 2011a). An alternative explanation is that the nodules form at focal points where ameloblasts overproduce matrix proteins and “swell out” to keep these areas covered.

At section levels 4 and 5, the deeply stained material covering the outer enamel becomes a continuous third layer of uneven thickness between the outer enamel and the maturation stage ameloblasts (Fig. S8). This layer becomes progressively less‐stained and forms an uneven crust over the outer enamel. The weakness of the enamel layer is highlighted at section level 9 where it appears to have separated from the underlying dentin (presumably being unable to sustain the shear forces associated with eruption or occlusion). Remarkably, cells from the overlying enamel organ seem to have moved through the crack and bridged the space between dentin and the inner enamel, indicating that the break was not an artifact of preparation, but actually occurred prior to this portion of the tooth erupting into the oral cavity. The *Mmp20* ^−/−^ *Klk4* ^−/−^ mice (Fig. S9) showed similar histology to the *Mmp20* ^−/−^ single null, except that the third layer did not seem to mineralize as readily and the separation tended to occur between the second and third layers (above the outer enamel). The third layer did not mineralize as quickly in the double null relative to the *Mmp20* ^−/−^ single null mice. Enamel formation in *Klk4* ^+/−^ (Fig. S10), *Mmp20* ^+/−^ (Fig. S11), and *Mmp20* ^+/−^ *Klk4* ^*+/−*^ (Fig. S12) mice appeared to be identical to the wild‐type, with the lone exception that staining of the enamel matrix sometimes persisted through to level 7, suggesting a minor delay in the removal of organic matrix in the double heterozygous mice.

**Figure 8:**
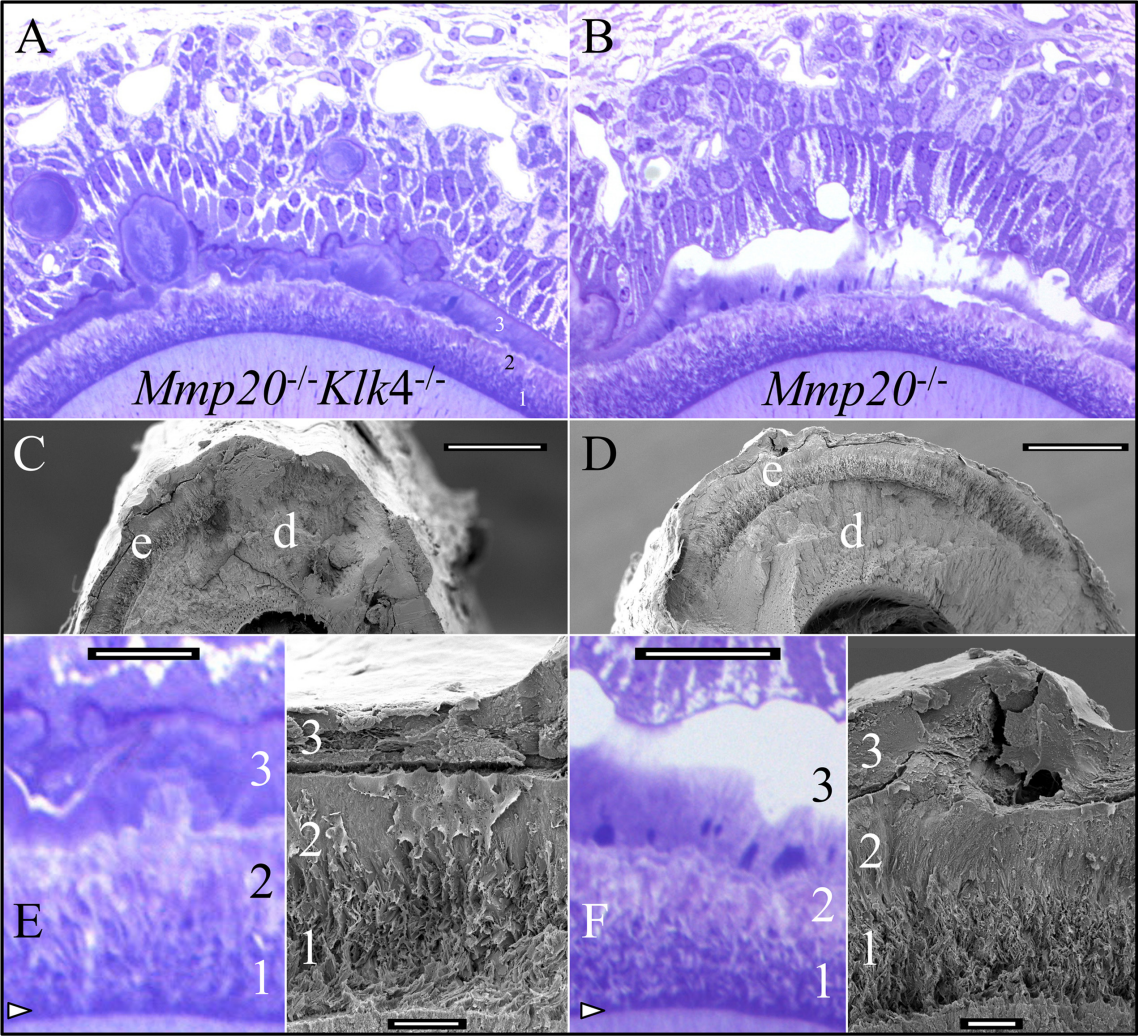
*Mmp20* ^*−/−*^ *Klk4* ^−/−^ and *Mmp20* ^*−/−*^ mandibular incisors at 7 weeks. (A–B) Histology of mandibular incisors at level 7. The enamel layer in both mice is very similar and divided into 3 layers. One principle difference is that the most superficial part of layer 3 does not stain in the *Mmp20* single nulls. It appears to be more highly mineralized and lower in protein. (C–D) Scanning electron microscopy( SEM) images at level 8 showing the three mineral layers covering dentin. Bars = 100 *μ*m. (E–F) Higher magnification views of the histology at level 7 and SEM images from level 8. Bars = 10 *μ*m. The dentino‐enamel junction (DEJ) (arrowheads) is highly stained and poorly mineralized. The first two layers, although thin and pathological, form during the secretory stage and correspond to the inner and outer enamel. The third layer has no corresponding structure in normal enamel, mineralizes during the maturation stage, and appears to become more highly mineralized when KLK4 is expressed. Key: d, dentin; e, enamel.

Scanning electron microscopy (SEM) images of the manibular incisors deliberately fractured at the level of the alveolar crest and histological images of analogous *Mmp20* ^*−/−*^ and *Mmp20* ^*−/−*^ *Klk4* ^−/−^ incisor cross sections were compared (Fig. 8). Histologically, the DEJ was distinct and deeply stained, reflecting its high protein content and low degree of mineralization. The inner (1) and middle (2) layers of enamel were readily distinguished histologically by the deeper staining of the inner layer. The surface layer (3) was continuous with mineral nodules in the soft tissue. This third layer stained strongly in MK double null mice, whereas the superficial portion appeared to be highly mineralized and largely cleared of protein in *Mmp20* ^−/−^ mice. These differences indicate that the cells in contact with the surface of layer 3 were still behaving like maturation stage ameloblasts, resorbing protein, and promoting mineralization. The third layer readily separated from the second.

## Discussion

Dental enamel formation occurs by a biologic mechanism that originated in lobe‐finned fish with lungs some 400–450 million years ago (Kawasaki et al. 2009). The key innovation was replacing the basal lamina with a mineralization front apparatus that deposits thin mineral ribbons on the incipient surface of dentin and extends them to the future enamel surface (Simmer et al. 2012a). The formation of these mineral ribbons is highly conserved and still remarkably similar in lungfish and mammals (Satchell et al. 2000; Ronnholm 1962a). Electron diffraction of the highly oriented, thin mineral ribbons near the mineralization front indicate that they are comprised of amorphous calcium phosphate (Beniash et al. 2009), or very poorly crystalline hydroxyapatite (Landis et al. 1988). These findings contradict the major classical mechanism for enamel formation that attempts to explain the novel ribbon morphology of enamel crystals by postulating an octacalcium phosphate precursor phase and the specific binding of acidic protein inhibitors onto selected crystal faces, which are subsequently removed by proteolysis (Simmer and Fincham 1995). As the enamel ribbons take their shape before they are crystalline, specific interactions with selected faces are impossible and the ribbon shape is not intrinsic to the mineral itself, but must somehow be imposed upon it externally, possibly by passing mineral through a shaped opening or into a mold. It is also observed that the thickness of the crystallites progressively increases with increasing distance from the ameloblast layer (Ronnholm 1962b), which should not occur in the presence of bound inhibitors. The demise of the classical theory requires a fresh analysis of the roles of proteases in the formation of dental enamel.

Although MMP20 is expressed by both odontoblasts and ameloblasts, MMP20 is not necessary for normal dentinogenesis. No dentin malformations are observed in *Mmp20* null mice or in people with AI caused by defects in *MMP20*. Furthermore, *Mmp20* has been pseudogenized in the sloth and aardvark, enamelless mammals that still make dentin (Meredith et al. 2014). So if the expression of MMP20 by odontoblasts is important, it must be in the formation of the DEJ. MMP20 is absolutely critical for proper formation of the DEJ, as the enamel fractures at the DEJ in *Mmp20* null mice (Simmer et al. 2012a). The initial mineralization of dentin generates an irregular mineral surface that is below the surface of the predentin matrix, so that frayed ends of collagen fibrils radiate out from the mineral toward the ameloblast membrane (Arsenault and Robinson 1989; Kallenbach 1976). Enamel proteins and MMP20 are secreted from ameloblast processes onto the freshly mineralized dentin surface and enamel mineral ribbons appear suddenly, associated with both the dentin crystals on one end and with the ameloblast membrane on the other. Although the enamel crystallite orientation is less regular in the contact area (Ronnholm 1962b), the mineral ribbons quickly orient at right angles to both the surface of the dentin and the ameloblast membrane (Reith 1967). The c‐axes of the dentin and enamel hydroxyapatite (HAP) crystals are oriented in the long axes of both the collagen fibrils and the enamel ribbons, respectively. As the collagen fibrils and the enamel ribbons run in the same direction and are closely associated (Fang et al. 2011), it is plausible that the dentin hydroxyapatite crystals might influence the orientation of the crystal lattice within the mineral ribbons, but not the orientation of the ribbons themselves, which are oriented by the mineralization front at the ameloblast membrane. Transmission electron microscopy (TEM) analyses have demonstrated that the initial enamel ribbons adjacent to mantle dentin form normally in *Mmp20* null mice (Beniash et al. 2006), so the absence of MMP20 proteolytic activity is not necessary for shaping the enamel ribbons, but results in a weaker bonding between dentin and enamel crystals. The weakness at the DEJ is so severe that the enamel layer shears off dentin at a level where the incisor that has not even erupted out of the soft tissue, and cells are able to invade the cracks to cover the exposed dentin.

In contrast to MMP20, KLK4 is never expressed by odontoblasts and is not expressed by ameloblasts until the enamel layer has reached full‐thickness—long after formation of the DEJ. However, in the *Klk4* null mouse the failure of proteins to return to the enamel surface for reabsorption by maturation stage ameloblasts increases with depth, so the enamel is weakest just above the DEJ and tends to fracture there (Simmer et al. 2009a). The bases of the enamel ribbons in the inner enamel mature significantly (grow in width and thickness) during the secretory stage, and the space required for this expansion is gained by the reabsorption of amelogenin cleavage products generated by MMP20 (Fukae et al. 2007). Continued maturation of the enamel crystals, especially the part of the crystals in the inner enamel, depends upon the removal of proteins in the spaces between crystals, which in turn depends upon KLK4 activity (Smith et al. 2011b). This partial overlapping of MMP20 and KLK4 functions likely explains the observed enamel failures (attrition) in *Mmp20Klk4* double heterozygous mice, which occurred in the deeper enamel. To our knowledge this is the first demonstration of enamel malformations caused by a digenic effect, which has implications on the types of strategies required to diagnose the genetic etiology in patients with AI.

Although critical for formation of the DEJ, MMP20 is also required for subsequent events during the secretory stage. The tips of the enamel ribbons are always associated with a “mineralization front” where enamel proteins are secreted. The mineral ribbons abut against a relatively smooth mineralization front that is attached to a cell membrane that is both undulated and infolded (Nanci and Warshawsky 1984). There are two similar, but spatially distinct secretory sites associated with the formation of rod and interrod enamel (Nanci and Warshawsky 1984). The complexity of the mineralization front in part reflects the simultaneous secretion, cleavage and reabsorption of proteins, exchange of ions, and cell movement that results in the precise deposition of calcium phosphate mineral onto the tips of ~10,000 enamel ribbons per ameloblast, each having cross‐sectional dimensions of ~1.5 nm x ~15 nm and separated from each other by a much larger area of organic matrix (Daculsi and Kerebel 1978; Ronnholm 1962b), possibly comprised of amelogenin nanospheres (Fincham et al. 1994, 1995). Enamelin (Fukae et al. 1996; Hu et al. 1997c), and ameloblastin (Krebsbach et al. 1996; Cerny et al. 1996; Hu et al. 1997b) are essential mineralization front components as no enamel ribbons form in *Enam* or *Ambn* knockout mice (Hu et al. 2008; Fukumoto et al. 2004; Smith et al. 2009; Hu et al. 2014), whereas a thinner than normal layer of defective enamel containing characteristic enamel mineral ribbons forms in *Amelx* and *Mmp20* null mice (Gibson et al. 2001; Caterina et al. 2002; Beniash et al. 2006). Amelogenin, enamelin, and ameloblastin are all rapidly cleaved by MMP20 following their secretion. Most of the amelogenin protein accumulates, whereas only smaller fragments from the N‐terminal or near N‐terminal regions of ameloblastin and enamelin are retained in the extracellular matrix (Nanci et al. 1998; Uchida et al. 1997, 1991; Tanabe et al. 1990b). Amelogenin is less abundant at the mineralization front where the enamel ribbons are shaped and oriented than it is in the bulk enamel beneath the mineralization front (Uchida et al. 1989; Nanci et al. 1998). This distribution is more consistent with a role in supporting the thin ribbons than in shaping and orienting them, which occurs the absence of amelogenin but is not sustained, resulting in an enamel layer that is much thinner than normal in *Amelx* null mice.

A major feature of *Mmp20* null enamel is the pathological accumulation of abundant extracellular matrix between and under ameloblasts that seems to flow onto the surface of the previously deposited enamel (layer 2) or accumulate near the cervical margins. In some cases it appears to flow through channels between cells and generate tubular or spherical nodules on the enamel surface. The matrix that mineralizes into this third layer is deposited more incisally than is normally covered by secretory stage ameloblasts, but the cells associated with its deposition resemble secretory stage ameloblasts. This finding suggests that in the absence of MMP20 there is a delay in transition from secretory to maturation stage or an increased eruption rate, or both. This third layer, being at the surface, undergoes a maturation processes that allows it to become more highly mineralized than the inner enamel layers. This is consistent with our determination that the enamel layer produced by *Mmp20* null mice has significantly more high‐density mineral than is the case for *Mmp20Klk4* double null mice where maturation stage activities are compromised.

We speculate that the organic matrix of the outer enamel layer formed in the *Mmp20* null enamel might relate to an obscure feature of normal amelogenesis, where large patches or droplets of amorphous electron‐dense material accumulate between adjacent rows of secretory stage ameloblasts and, to a lesser extent, between ameloblasts within rows (Nanci and Warshawsky 1984). This intercellular material presumably empties into the enamel matrix at the interrod grow sites by transient, controlled interruptions in the distal junctional complex. Disruption of this mechanism in *Mmp20* null mice suggests that MMP20 cleavages are necessary to drain the droplets of intercellular organic material into the matrix, perhaps to stiffen it in support of ameloblast retrograde movements that allow for subsequent thickening of the enamel layer. This scenario is consistent with hypotheses that MMP20 facilitates ameloblast movement by cleaving ameloblast cell–cell contacts (Guan and Bartlett 2013).

The enamel malformations observed in the absence of MMP20, KLK4, or both enzymes provide strong evidence that the function of secreted enamel proteases is not to cleave crystal‐bound acidic proteins that inhibit mineral deposition on select crystal faces so that they can grow in width and thickness after adopting an elongated form. MMP20 might cleave inhibitors that prevent the solid attachment of the initial enamel ribbons to dentin, but plays no role in shaping the enamel ribbons. MMP20 catalyzes all of the cleavages of enamel proteins that occur during the secretory stage. These cleavages balance continued protein secretion with the reabsorption of selected cleavage products, which sustains the rhythmic, cyclical elongation of enamel ribbons at the mineralization front and the accumulation of cleavage products that support and separate the thin mineral ribbons, but must also be progressively degraded and reabsorbed to provide space for continuous growth of the thin crystallites in width and thickness. The complexity of the enamel phenotype displayed in the absence of MMP20 encourages the belief that MMP20 likely catalyzes cleavages in proteins besides amelogenin, enamelin, and ameloblastin that influence cellular processes that are at the present time, poorly understood.

KLK4 is secreted by maturation stage ameloblasts to degrade enamel proteins to facilitate their diffusion to the enamel surface for removal by endocytosis. The maturation of enamel takes less than 2 weeks in rodents where the enamel layer is only 115–120 *μ*m thick. In humans, where the enamel layer can grow to more than 2 mm in thickness, the process can take up to 5 years to complete. Thus, there is a trade‐off between enamel thickness and the amount of time required for enamel maturation (Smith 1998). KLK4 is a relatively recent evolutionary innovation that facilitates the removal of enamel matrix. Without KLK4, there is a problem removing enamel proteins that increases in severity with distance from the enamel surface. Enamel crystals need to grow together and interlock with adjacent crystals, or they fail during function. Both MMP20 and KLK4 facilitate the removal of enamel proteins, and both activities are critically important for removing proteins in the inner enamel. As a consequence of this overlapping function, failure of the enamel occurs in the *Mmp20Klk4* double heterozygous mice, but not in the *Mmp20* or *Klk4* single heterozygotes. MMP20 and KLK4 also serve complementary functions so that enamel formed in the *Mmp20Klk4* double null mice does not contain as much high‐density enamel as it does in either the *Mmp20* or *Klk4* single nulls. Despite significant progress in our understanding of the mechanisms of enamel biomineralization and the roles of secreted proteases in the process, we have no explanation for the prominent, evenly spaced horizontal ridges observed in the mandibular incisors from the *Mmp20* null and *Mmp20Klk4* double null mice.

## Conflict of Interest

None declared.

## Supporting information


**Figure S1.** PCR Genotyping.
**Figure S2.** Cross sectioning a mandibular incisor.
**Figure S3.** Day 14 mandibular dentition prior to molar eruption as viewed under a dissecting microscope.
**Figure S4.** Day 17 mandibular dentition immediately following eruption of the first molar as viewed under a dissecting microscope.
**Figure S5.** Week 7 mandibular dentition as viewed under a dissecting microscope.
**Figure S6.** Week 7 wild‐type mandibular incisor cross sections.
**Figure S7.** Week 7 *Klk4* null mandibular incisor cross sections.
**Figure S8.** Week 7 *Mmp20* null mandibular incisor cross sections.
**Figure S9.** Week 7 *Mmp20*/*Klk4* double null mandibular incisor cross sections.
**Figure S10.** Week 7 *Klk4* heterozygous mandibular incisor cross sections.
**Figure S11.** Week 7 *Mmp20* heterozygous mandibular incisor cross sections.
**Figure S12.** Week 7 *Mmp20*/*Klk4* double heterozygous mandibular incisor cross sections.
**Figure S13.** Week 7 wild‐type maxillary incisor longitudinal section.
**Figure S14.** Week 7 wild‐type mandibular incisor longitudinal section.
**Figure S15.** Week 7 *Klk4* null maxillary incisor longitudinal section.
**Figure S16.** Week 7 *Klk4* null mandibular incisor longitudinal section.
**Figure S17.** Week 7 *Mmp20* null maxillary incisor longitudinal section.
**Figure S18.** Week 7 *Mmp20* null mandibular incisor longitudinal section.
**Figure S19.** Week 7 *Mmp20* ^−/−^ *Klk4* ^−/−^ maxillary incisor longitudinal section.
**Figure S20.** Week 7 *Mmp20* ^−/−^ *Klk4* ^−/−^ mandibular incisor longitudinal section.
**Figure S21.** Week 7 *Klk4* heterozygous maxillary incisor longitudinal section.
**Figure S22.** Week 7 *Klk4* heterozygous mandibular incisor longitudinal section.
**Figure S23.** Week 7 *Mmp20* heterozygous maxillary incisor longitudinal section.
**Figure S24.** Week 7 *Mmp20* heterozygous mandibular incisor longitudinal section.
**Figure S25.** Week 7 *Mmp20*/Klk4 double heterozygous maxillary incisor longitudinal section.
**Figure S26.** Week 7 *Mmp20*/Klk4 double heterozygous mandibular incisor longitudinal section.
