# Supplementary material for: MMP20, KLK4, and MMP20/KLK4 double null mice define roles for matrix proteases during dental enamel formation
Source: Mol Genet Genomic Med. 2015 Dec 20;4(2):178–96. doi: 10.1002/mgg3.194 (PMC4799876; doi:10.1002/mgg3.194)
Supplement: Supplementary file 1 — Figure S1. PCR Genotyping. Figure S2. Cross sectioning a mandibular incisor. Figure S3. Day 14 mandibular dentition prior to molar eruption as viewed under a dissecting microscope. Figure S4. Day 17 mandibular dentition immediately following eruption of the first molar as viewed under a dissecting microscope. Figure S5. Week 7 mandibular dentition as viewed under a dissecting microscope. Figure S6. Week 7 wild‐type mandibular incisor cross sections. Figure S7. Week 7 Klk4 null mandibular incisor cross sections. Figure S8. Week 7 Mmp20 null mandibular incisor cross sections. Figure S9. Week 7 Mmp20/Klk4 double null mandibular incisor cross sections. Figure S10. Week 7 Klk4 heterozygous mandibular incisor cross sections. Figure S11. Week 7 Mmp20 heterozygous mandibular incisor cross sections. Figure S12. Week 7 Mmp20/Klk4 double heterozygous mandibular incisor cross sections. Figure S13. Week 7 wild‐type maxillary incisor longitudinal section. Figure S14. Week 7 wild‐type mandibular incisor longitudinal section. Figure S15. Week 7 Klk4 null maxillary incisor longitudinal section. Figure S16. Week 7 Klk4 null mandibular incisor longitudinal section. Figure S17. Week 7 Mmp20 null maxillary incisor longitudinal section. Figure S18. Week 7 Mmp20 null mandibular incisor longitudinal section. Figure S19. Week 7 Mmp20 −/− Klk4 −/− maxillary incisor longitudinal section. Figure S20. Week 7 Mmp20 −/− Klk4 −/− mandibular incisor longitudinal section. Figure S21. Week 7 Klk4 heterozygous maxillary incisor longitudinal section. Figure S22. Week 7 Klk4 heterozygous mandibular incisor longitudinal section. Figure S23. Week 7 Mmp20 heterozygous maxillary incisor longitudinal section. Figure S24. Week 7 Mmp20 heterozygous mandibular incisor longitudinal section. Figure S25. Week 7 Mmp20/Klk4 double heterozygous maxillary incisor longitudinal section. Figure S26. Week 7 Mmp20/Klk4 double heterozygous mandibular incisor longitudinal section. [file MGG3-4-178-s001.pdf]

Supplemental Data

S1. PCR Genotyping

S2. Cross-sectioning a mandibular incisor

S3. Day 14 mandibular dentition prior to molar eruption as viewed under a dissecting microscope

S4. Day 17 mandibular dentition immediately following eruption of the first molar as viewed under a dissecting microscope

S5. Week 7 mandibular dentition as viewed under a dissecting microscope

S6. Week 7 wild-type mandibular incisor cross-sections

S7. Week 7 *Klk4* null mandibular incisor cross-sections

S8. Week 7 *Mmp20* null mandibular incisor cross-sections

S9. Week 7 *Mmp20*/*Klk4* double null mandibular incisor cross-sections

S10. Week 7 *Klk4* heterozygous mandibular incisor cross-sections

S11. Week 7 *Mmp20* heterozygous mandibular incisor cross-sections

S12. Week 7 *Mmp20*/*Klk4* double heterozygous mandibular incisor cross-sections

S13. Week 7 wild-type maxillary incisor longitudinal section

S14. Week 7 wild-type mandibular incisor longitudinal section

S15. Week 7 *Klk4* null maxillary incisor longitudinal section

S16. Week 7 *Klk4* null mandibular incisor longitudinal section

S17. Week 7 *Mmp20* null maxillary incisor longitudinal section

S18. Week 7 *Mmp20* null mandibular incisor longitudinal section

S19. Week 7 $Mmp20^{-/-}Klk4^{-/-}$ maxillary incisor longitudinal section

S20. Week 7 $Mmp20^{-/-}Klk4^{-/-}$ mandibular incisor longitudinal section

S21. Week 7 *Klk4* heterozygous maxillary incisor longitudinal section

S22. Week 7 *Klk4* heterozygous mandibular incisor longitudinal section

S23. Week 7 *Mmp20* heterozygous maxillary incisor longitudinal section

S24. Week 7 *Mmp20* heterozygous mandibular incisor longitudinal section

S25. Week 7 *Mmp20*/Klk4 double heterozygous maxillary incisor longitudinal section

S26. Week 7 *Mmp20*/Klk4 double heterozygous mandibular incisor longitudinal section

A

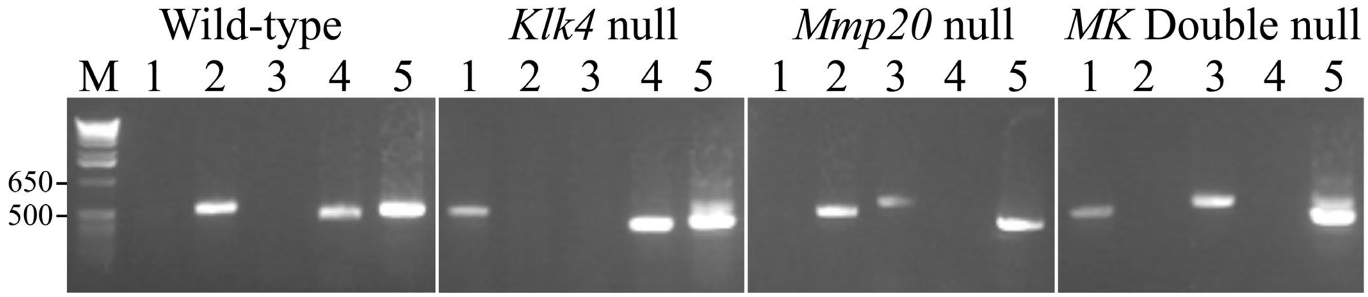

B

1. *Klk4-lac*Z primer pair[1] amplifies the *Klk4-lacZ* knockin gene (595 bp)
   94˚C 5'; [94˚C 30"; 58˚C 30"; 72˚C 1"]$^{35\ cycles}$; 72˚C 10'
   For: 5'TGCCTCCAACCAGATAGGTC Rev: 5'GACAGTATCGGCCTCAGGAA

2. *Klk4* Intron 3-Exon 5 primer pair[1] amplifies the *Klk*4 wild-type gene (550 bp)
   94˚C 5'; [94˚C 30"; 58˚C 30"; 72˚C 1"]$^{35\ cycles}$; 72˚C 10'
   For: 5'AACCTAAGGGACAGGGCAGT Rev: 5'TGAGGTGGTACACAGGGTCA

3. *Mmp20* p03 + p04 primer pair[2] amplifies the *Mmp20* knockout gene (670 bp)
   94˚C 5'; [94˚C 30"; 58˚C 30"; 72˚C 1"]$^{35\ cycles}$; 72˚C 10'
   For: 5'CTGCGTCCCCAGACTTTTGATTT Rev: 5'GCTTTTCATGGCCAGAATGCTCT

4. *Mmp20* p05 + p06 primer pair[2] amplifies the *Mmp20* wild-type gene (444 bp)
   94˚C 5'; [94˚C 30"; 58˚C 30"; 72˚C 1"]$^{35\ cycles}$; 72˚C 10'
   For: 5'AAGTAGACTGAAGTCAGGAGAGCC Rev: 5'CTGTAGTGGTGACCCTAGTCATCTT

5. A ß-*globin* primer pair is for genomic DNA quality control (494 bp)
   94˚C 5'; [94˚C 30"; 60˚C 90"; 72˚C 2"]$^{35\ cycles}$; 72˚C 10'
   For: 5'CCAATCTGCTCACACAGGATAGAGAGGGCAGG
   Rev: 5'CCTTGAGGCTGTCCAAGTGATTCAGGCCATCG

Figure S1. PCR Genotyping. ***A:*** 1% agarose gels stained with ethidium bromide. M is the 1 Kb Plus DNA ladder (Invitrogen, Carlsbad, CA). Numbered lanes correspond to PCR amplification products that test for the presence of the *Mmp20* or *Klk4* wild-type (WT) or knockout (KO) genes. Lanes: 1. *Klk4* KO, 2. *Klk4* WT, 3. *Mmp20* KO, 4. *Mmp20* WT, and 5. Reagent and DNA control. The positive lanes for each null genotype are: WT: 2, 4-5; *Klk4*$^{-/-}$: 4-5; *Klk4*$^{+/-}$: 1-2, 4-5; *MK* double heterozygote: 1-5; *MK* double null: 1, 3, 5. ***B:*** *Mmp20* [1] and *Klk4* [2] primers, amplification conditions, and expected product sizes for the 5 PCR reactions used for genotyping.

References

1. Caterina JJ, Skobe Z, Shi J, Ding Y, Simmer JP, Birkedal-Hansen H, Bartlett JD (2002) Enamelysin (matrix metalloproteinase 20)-deficient mice display an amelogenesis imperfecta phenotype. *J. Biol. Chem.* 277: 49598-49604.

2. Simmer JP, Hu Y, Lertlam R, Yamakoshi Y, Hu JC (2009) Hypomaturation enamel defects in Klk4 knockout/LacZ knockin mice. *J. Biol. Chem.* 284: 19110-19121.

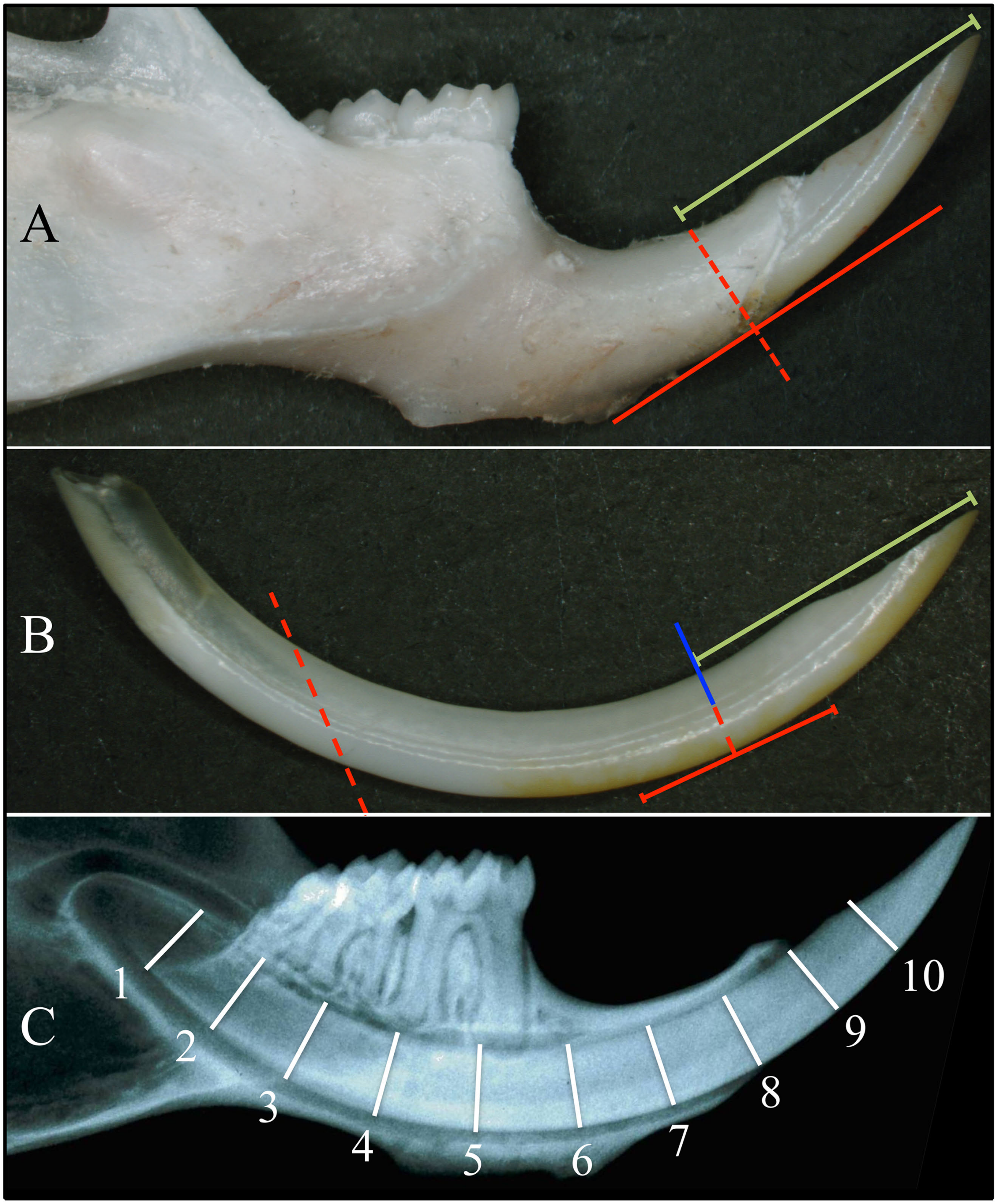

Figure S2. Cross-sectioning a mandibular incisor. Soft tissue is removed from the mandibles at 7-weeks. ***A:*** We measure the distances from the incisor tip to the labial alveolar crest (white line) and extract the incisors. ***B:*** On one incisor we use a rotating diamond disc (blue line) to cut through just the dentin (at the measured distance from the incisor tip, white line) and fracture the incisor at the notch (which highlights the decussating rods). The other incisor is cut all the way through with the disc, polished and lightly etched with 0.1% nitric acid. A second, parallel cut (red dashed line) is made through the incisors 5-6 mm apical to the first cut. This cut surface is glued to an SEM stub so that the electron beam is aimed directly perpendicular to the first cut to accurately measure the thickness of the enamel layer. The surface may be sputter coated with gold or not and analyzed by SEM or bSEM. **C:** Lines showing levels of cross-sections for histology.

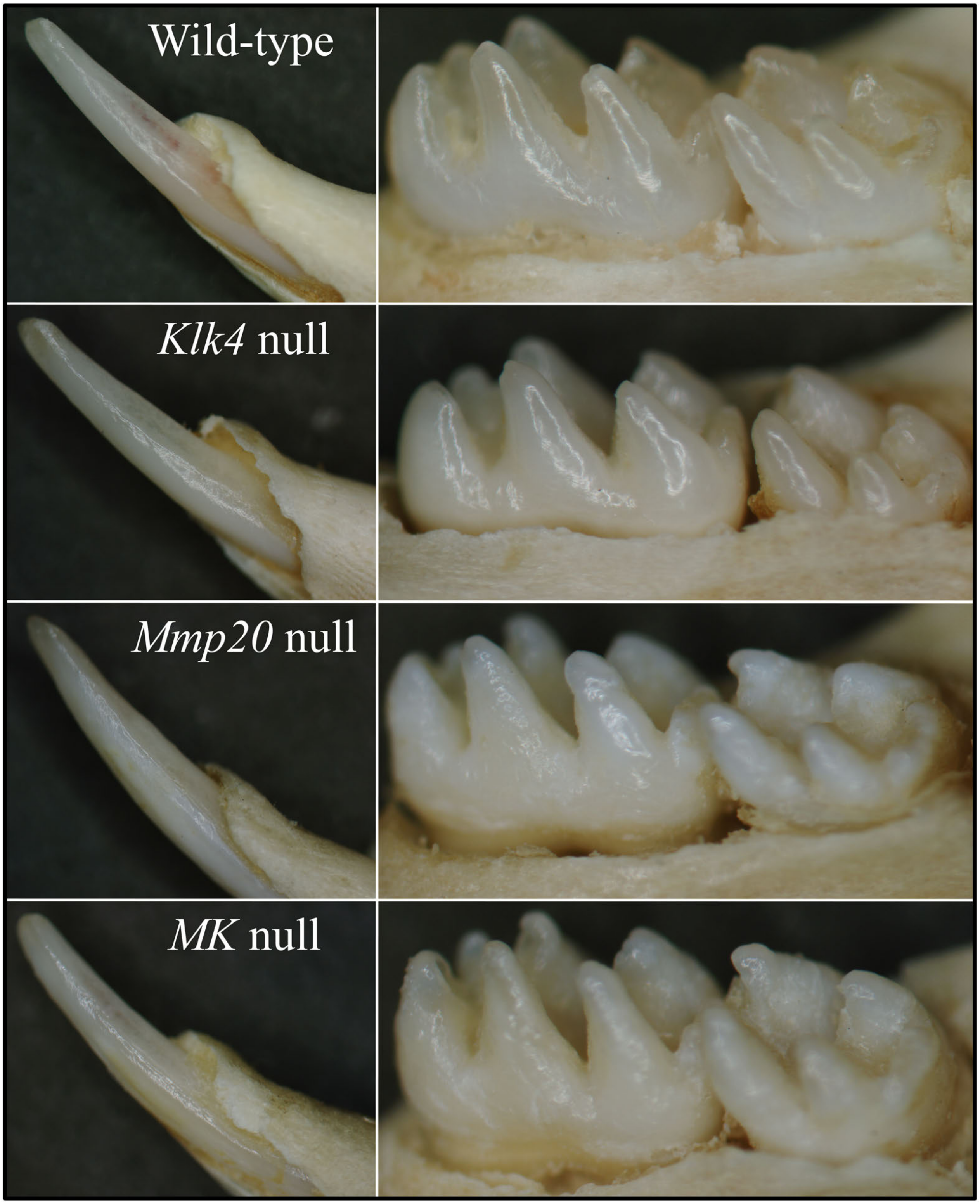

Figure S3. Day 14 mandibular dentition prior to molar eruption as viewed under a dissecting microscope. The wild-type and *Klk4* null dentitions are virtually indistinguishable prior to eruption. The *Mmp20* null enamel is similar to that of the *MK* double null, which is thin and rough-surfaced.

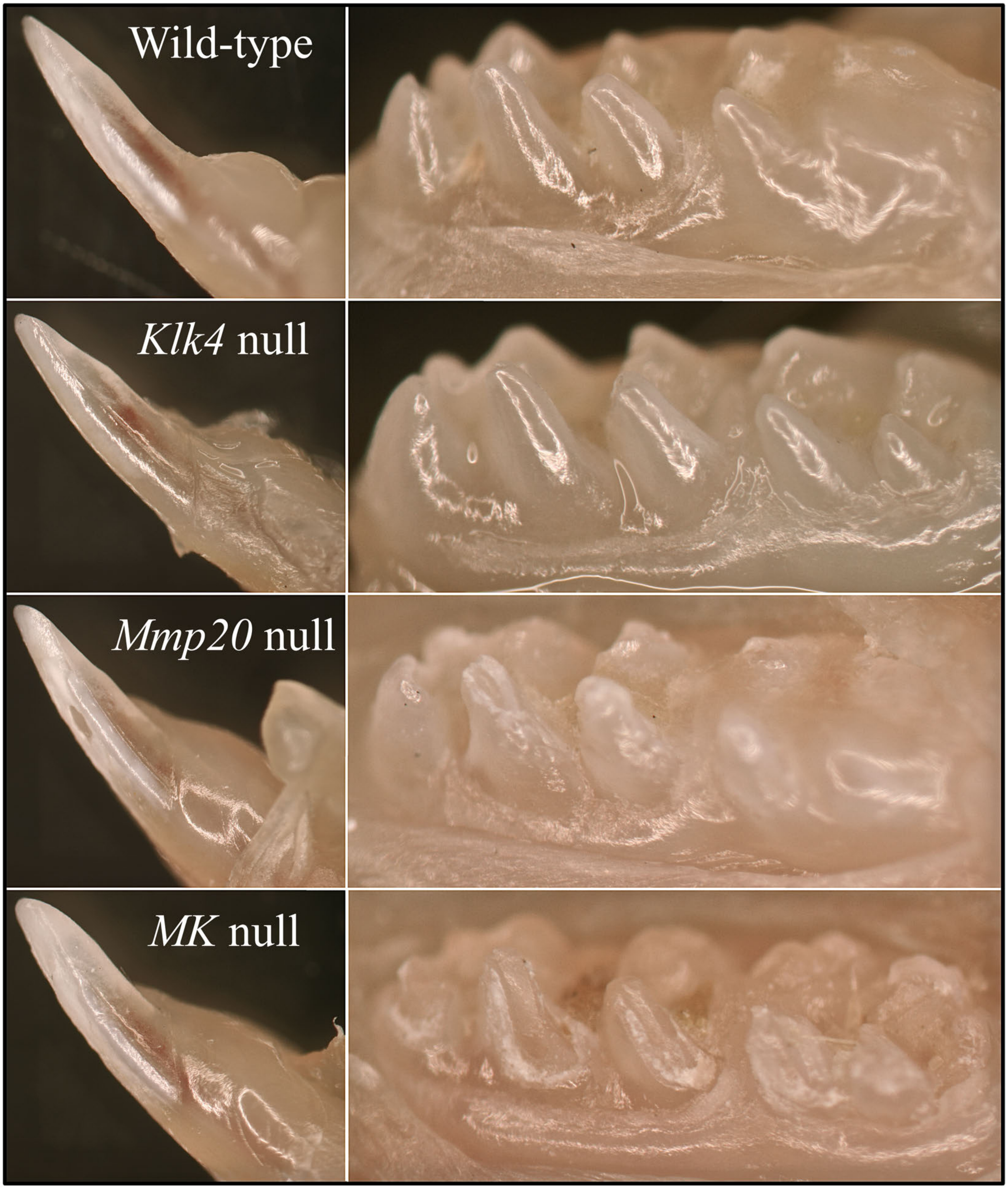

Figure S4. Day 17 mandibular dentition immediately following eruption of the first molar as viewed under a dissecting microscope. The *Mmp20* and *MK* double null enamel on the first molar already show signs of attrition on the buccal cusps.

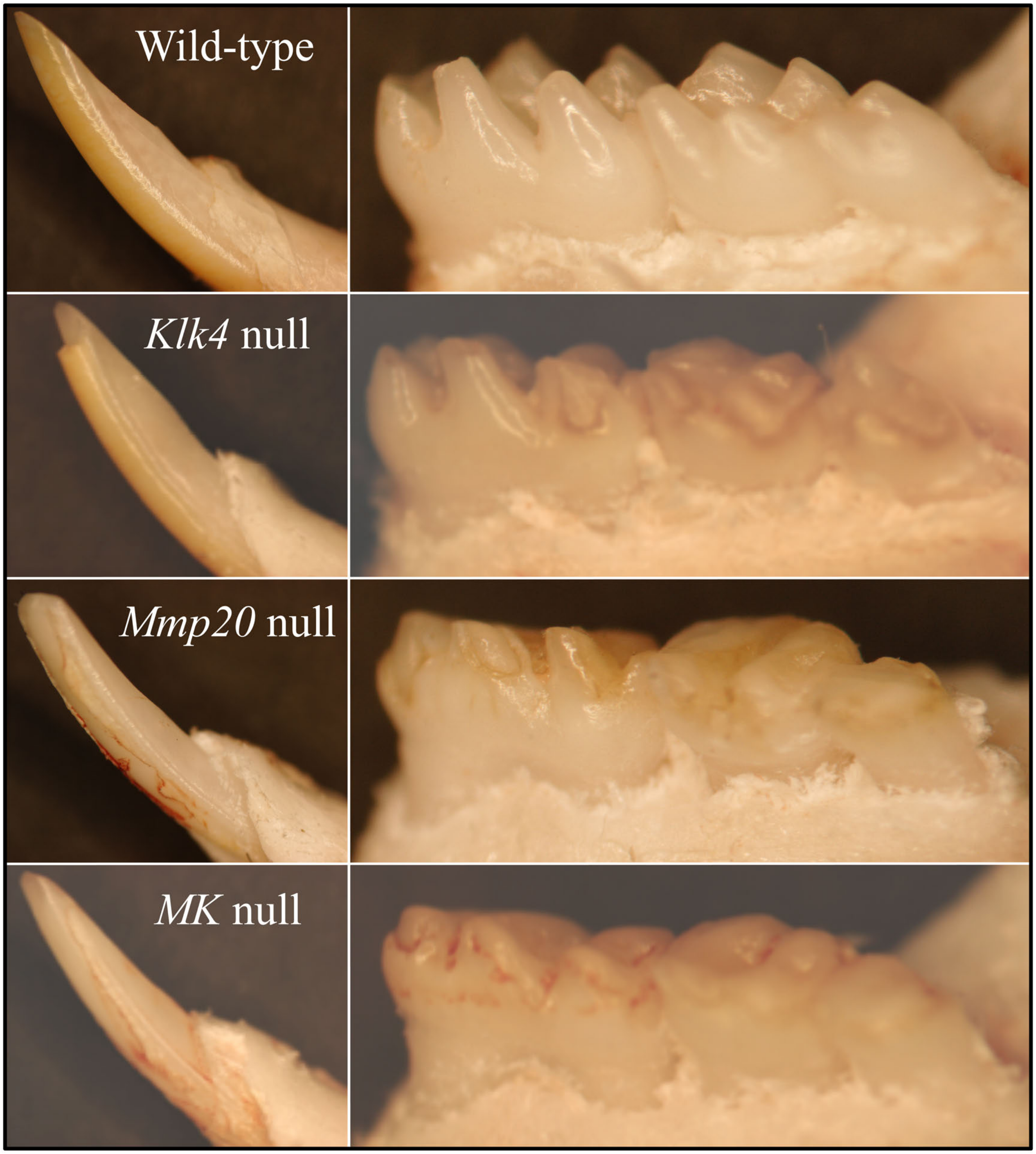

Figure S5. Week 7 mandibular dentition as viewed under a dissecting microscope. The *Klk4*, *Mmp20* and *MK* double null enamel show significant attrition on all teeth. The red color is an artifact of bleeding during soft tissue removal, but serves to highlight rough surfaces where the enamel has broken.

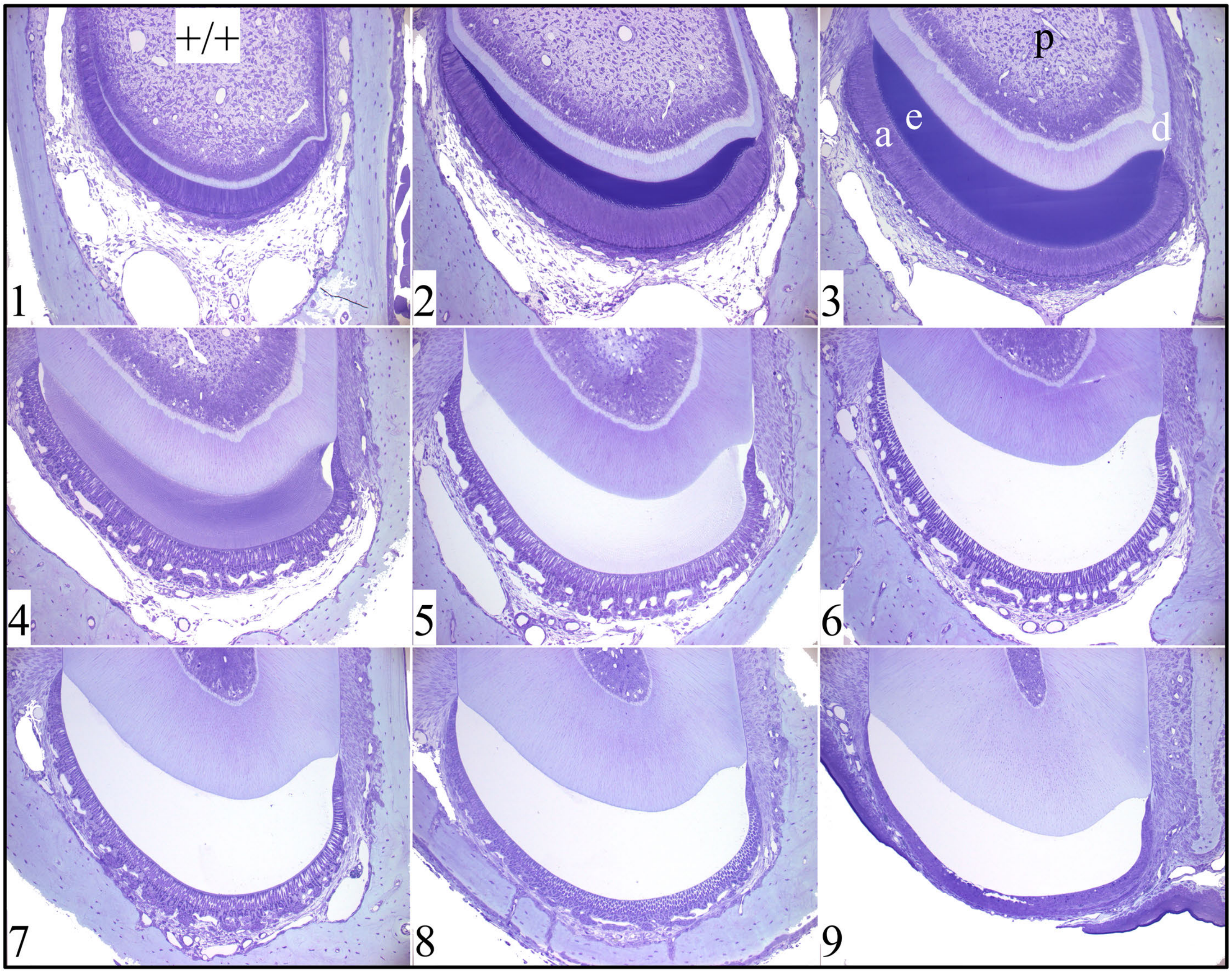

Figure S6. Week 7 wild-type mandibular incisor cross-sections. The sections were taken at 1 mm increments, at the approximate positions shown in Fig. S2C. The level 2 section shows the secretory stage of amelogenesis. The level 3 section shows late secretory stage or very early maturation. The level 4 section is all maturation stage. ***Key:*** a, ameloblasts; d, dentin; e, enamel; p, pulp.

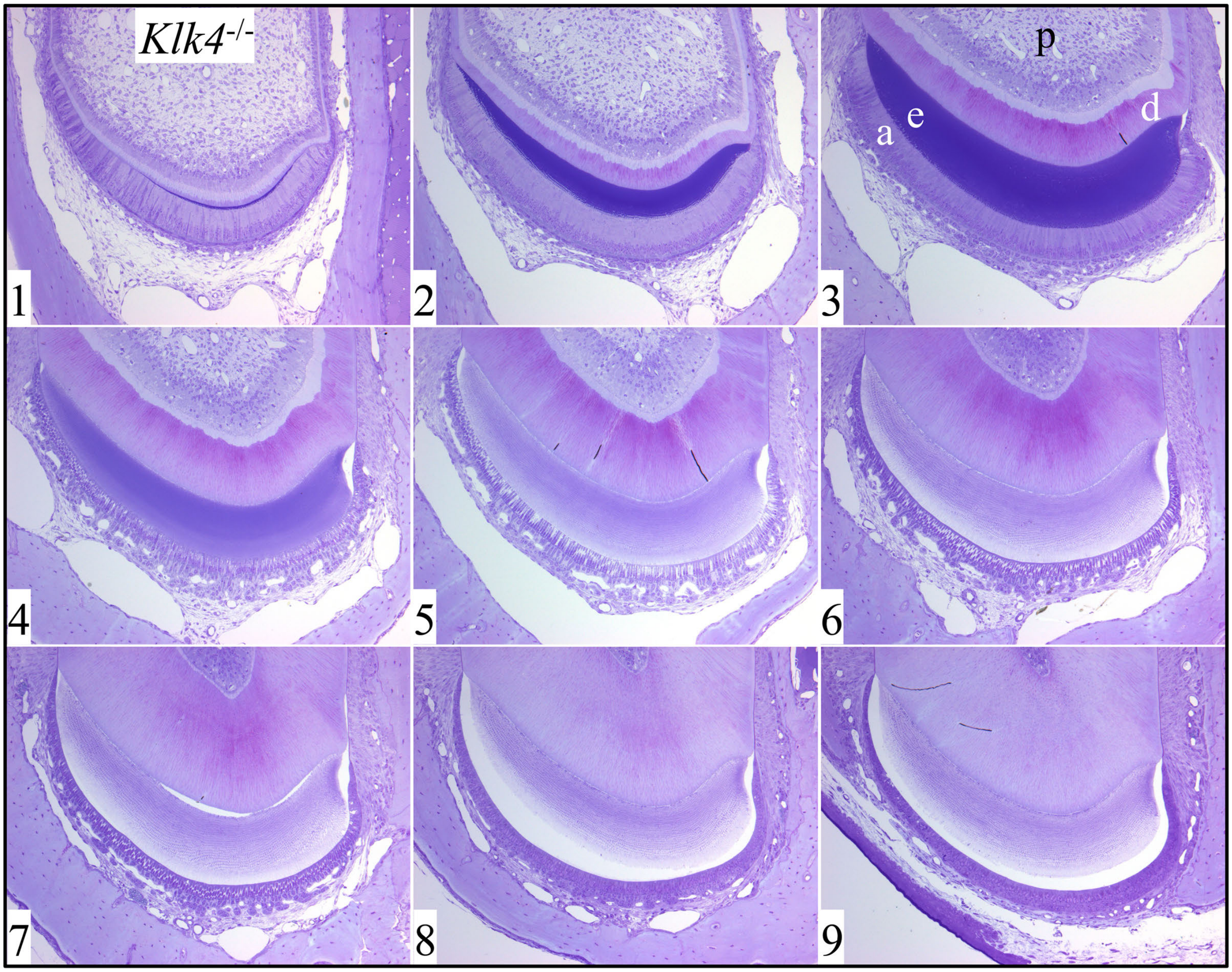

Figure S7. Week 7 *Klk4* null mandibular incisor cross-sections. The sections were taken at 1 mm increments, at the approximate positions shown in Fig. S2C. The level 2 section shows the secretory stage of amelogenesis. The level 3 section shows late secretory stage or very early maturation. The level 4 section is all maturation stage. ***Key:*** a, ameloblasts; d, dentin; e, enamel; p, pulp.

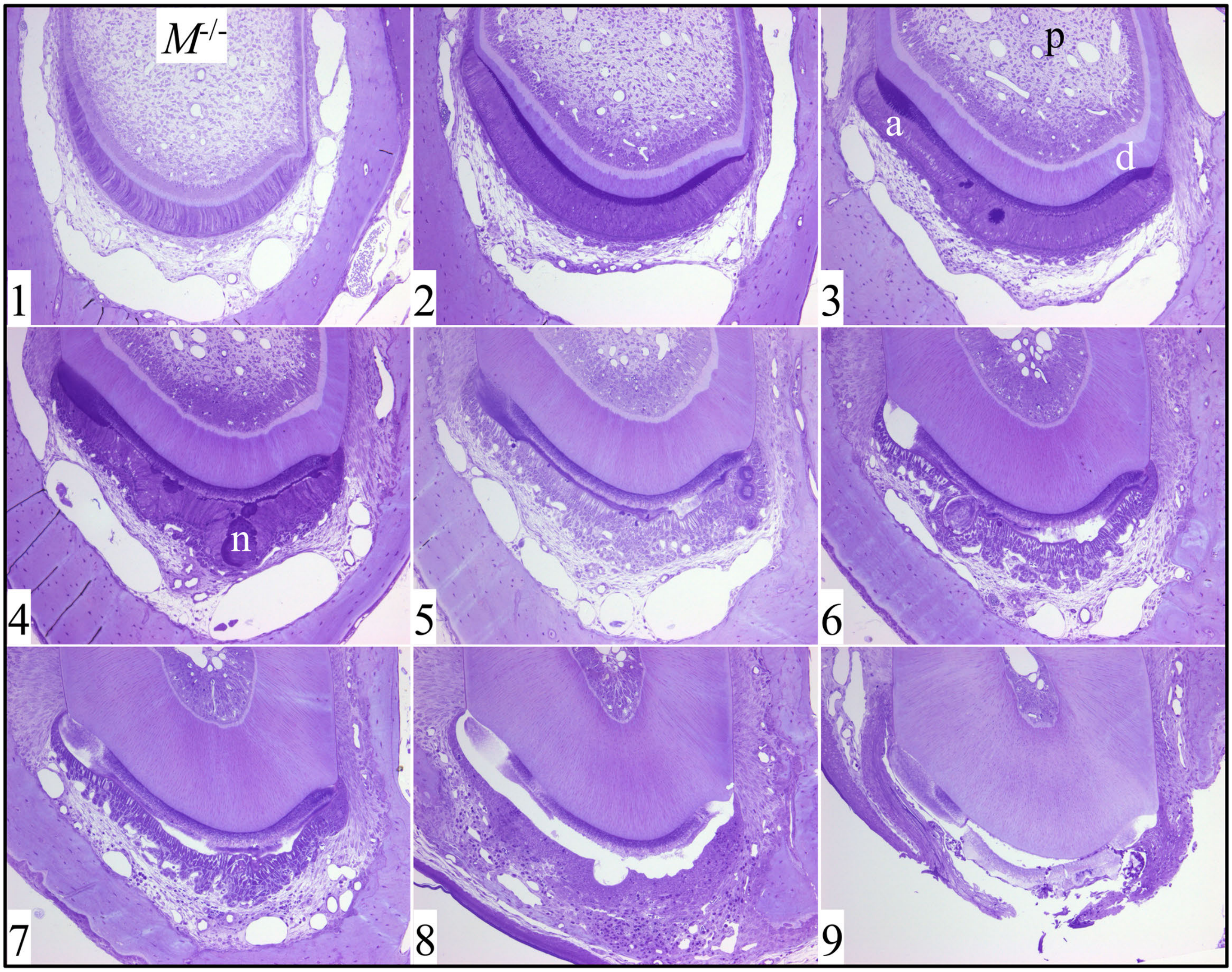

Figure S8. Week 7 *Mmp20* null mandibular incisor cross-sections. The sections were taken at 1 mm increments, at the approximate positions shown in Fig. S2C. The level 2 section shows the secretory stage of amelogenesis. The level 3 section shows late secretory stage or very early maturation. The level 4 section is all maturation stage. ***Key:*** a, ameloblasts; d, dentin; n, nodule; p, pulp.

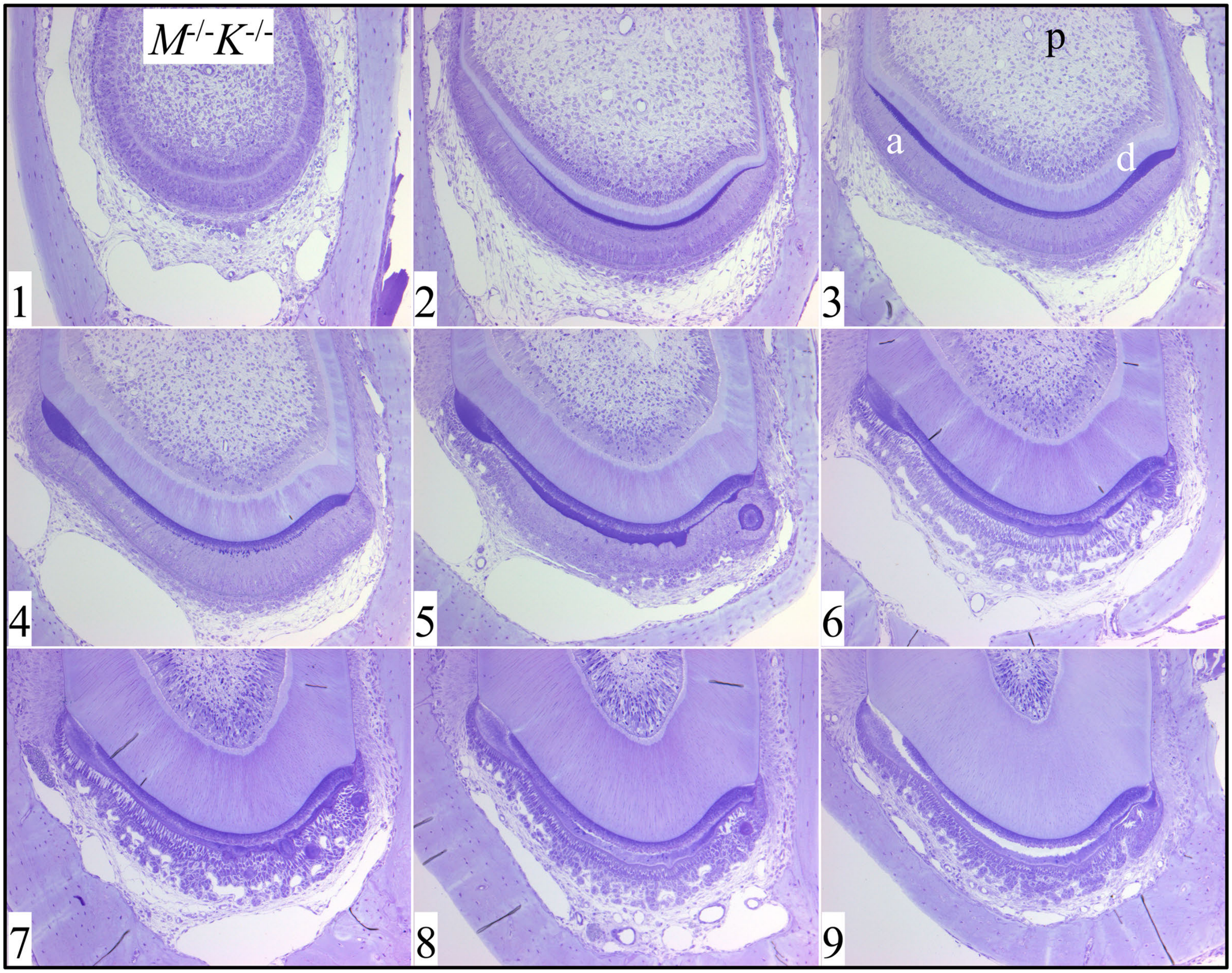

Figure S9. Week 7 *Mmp20/Klk4* double null mandibular incisor cross-sections. The sections were taken at 1 mm increments, at the approximate positions shown in Fig. S2C. The level 2 section shows the secretory stage of amelogenesis. The level 3 section shows late secretory stage or very early maturation. The level 4 section is all maturation stage. ***Key:*** a, ameloblasts; d, dentin; p, pulp.

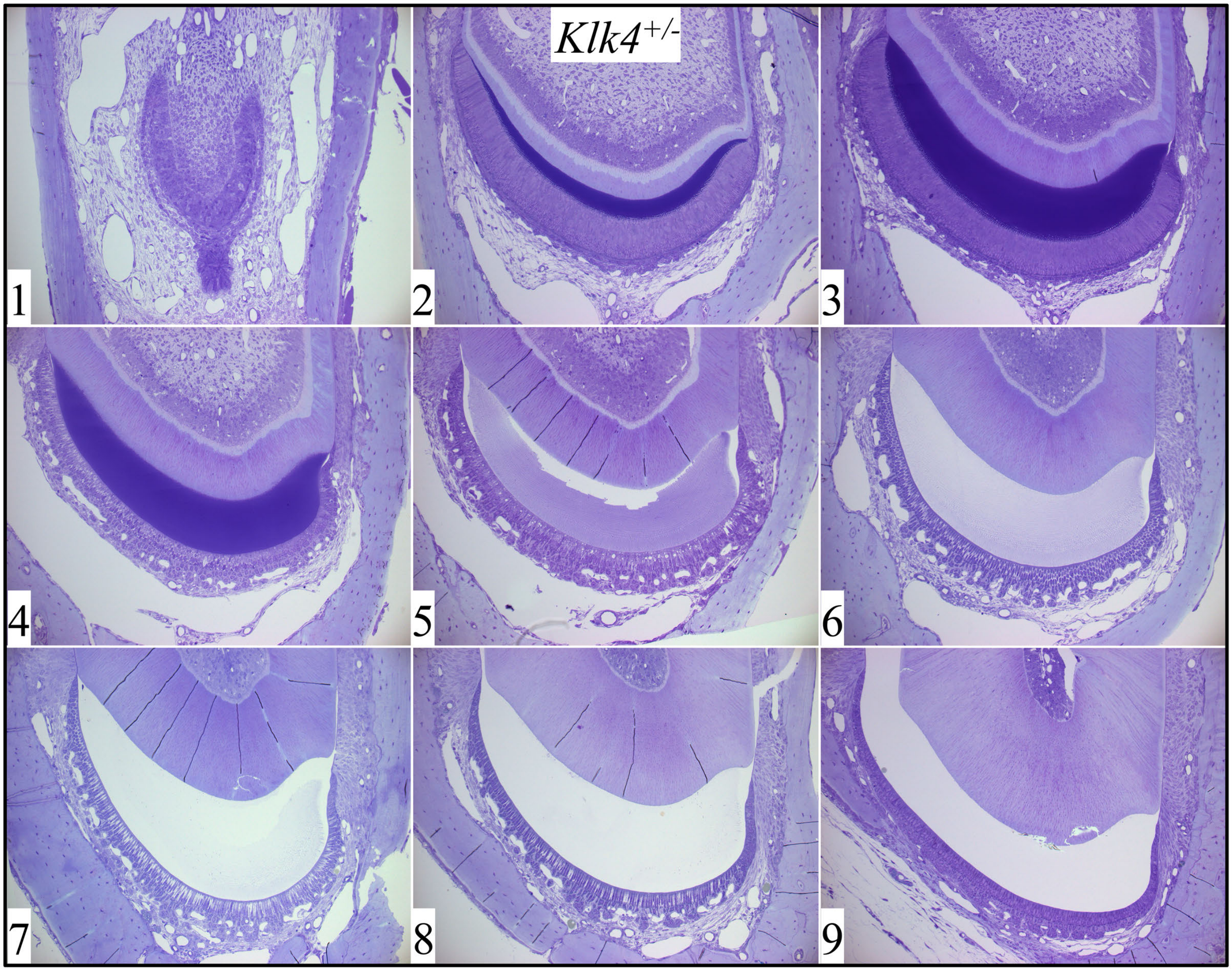

Figure S10. Week 7 *Klk4* heterozygous mandibular incisor cross-sections. The sections were taken at 1 mm increments, at the approximate positions shown in Fig. S2C. The level 2 section shows the secretory stage of amelogenesis. The level 3 section shows late secretory stage or very early maturation. The level 4 section is all maturation stage.

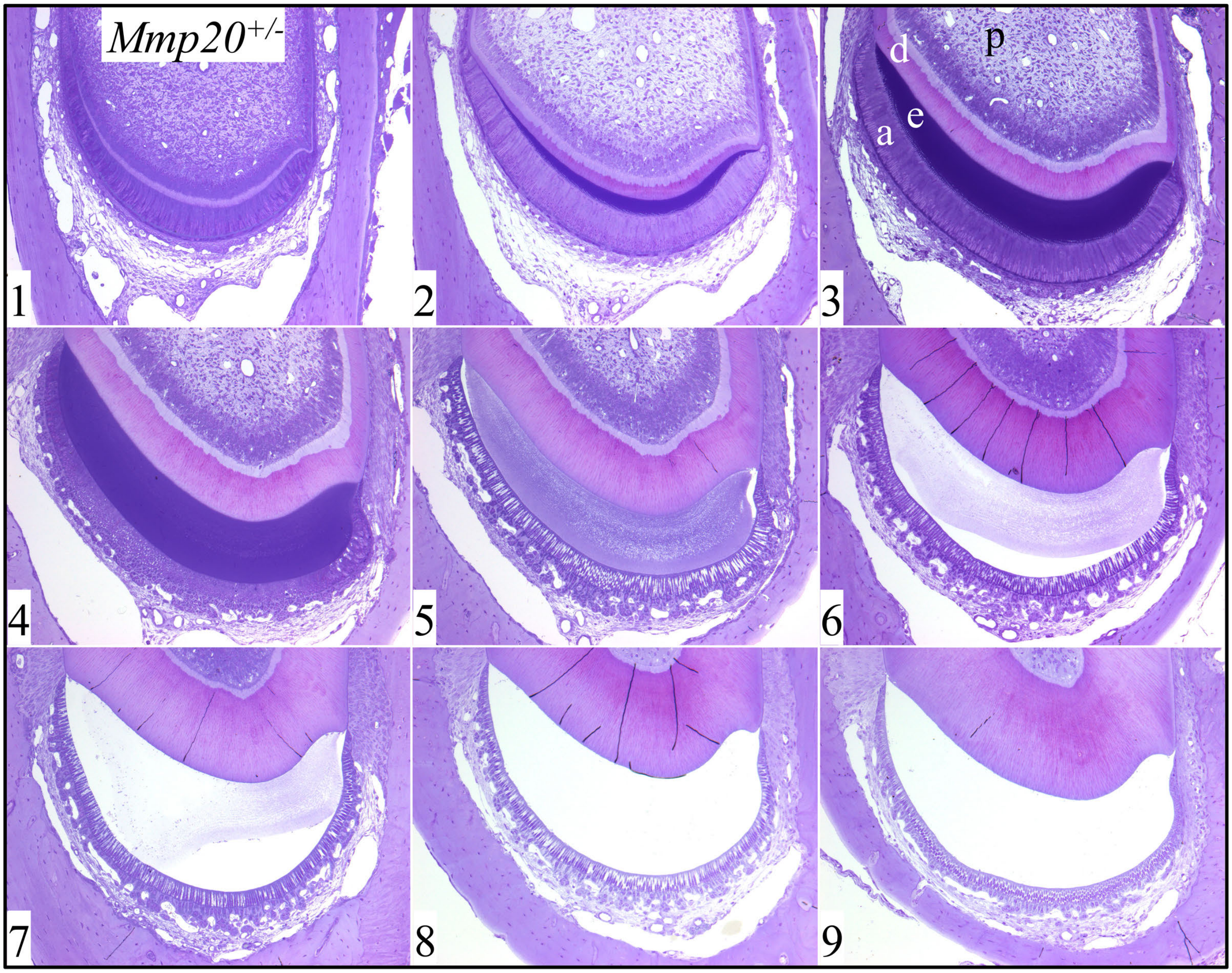

Figure S11. Week 7 *Mmp20* heterozygous mandibular incisor cross-sections. The sections were taken at 1 mm increments, at the approximate positions shown in Fig. S2C. The level 2 section shows the secretory stage of amelogenesis. The level 3 section shows late secretory stage or very early maturation. The level 4 section is all maturation stage. ***Key:*** a, ameloblasts; d, dentin; e, enamel; p, pulp.

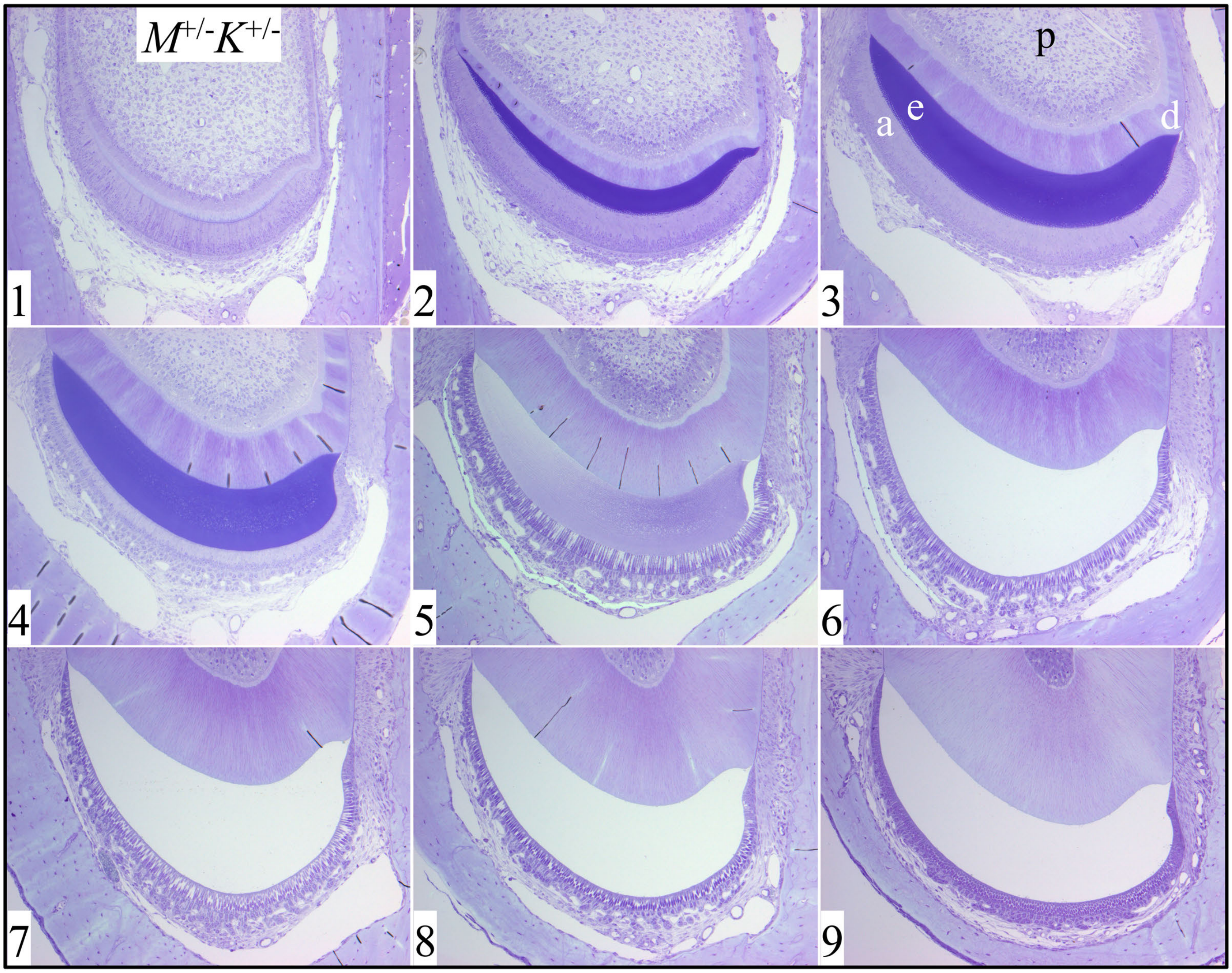

Figure S12. Week 7 *Mmp20/Klk4* double heterozygous mandibular incisor cross-sections. The sections were taken at 1 mm increments, at the approximate positions shown in Fig. S2C. The level 2 section shows the secretory stage of amelogenesis. The level 3 section shows late secretory stage or very early maturation. The level 4 section is all maturation stage. ***Key:*** a, ameloblasts; d, dentin; e, enamel; p, pulp.

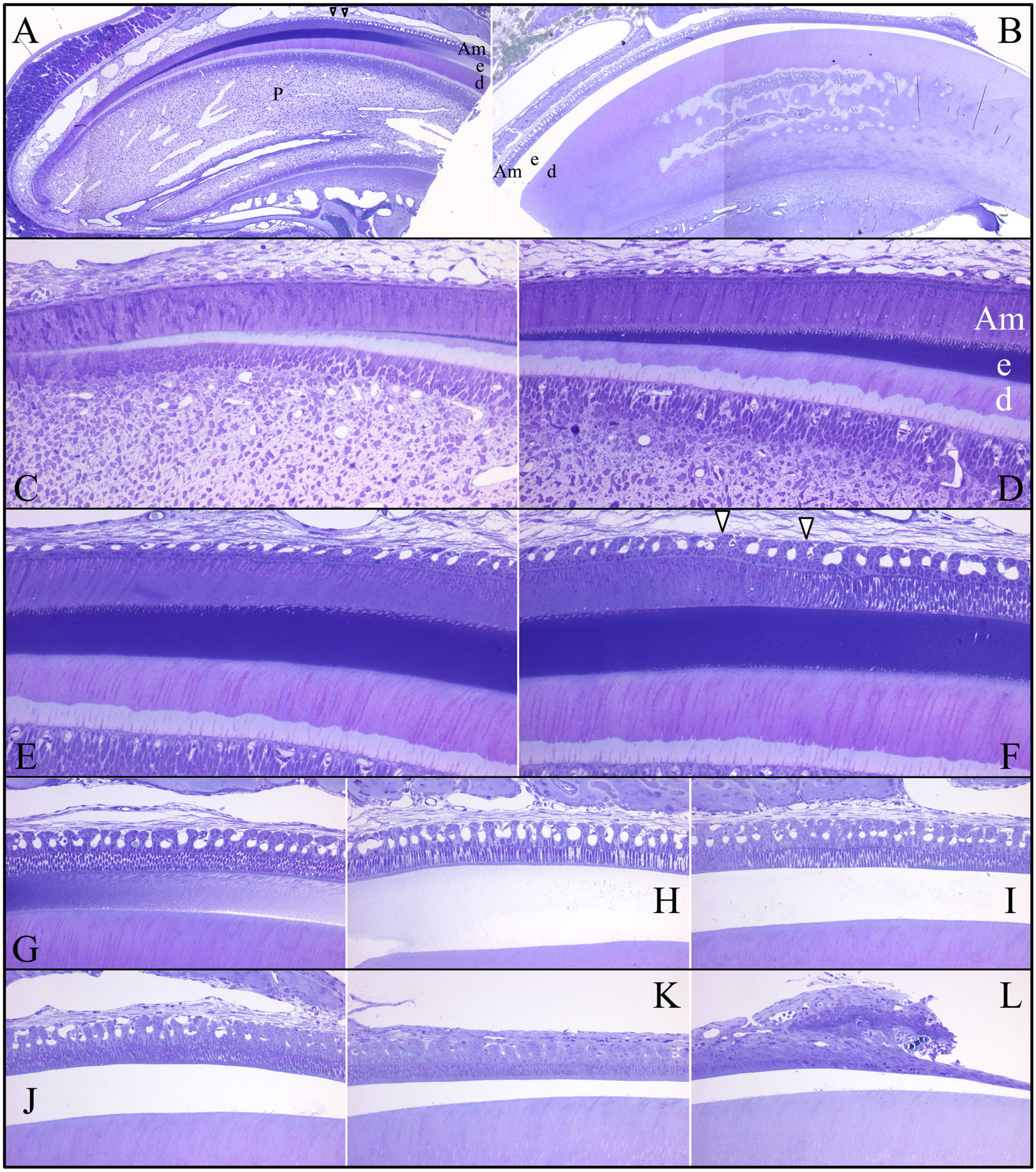

Figure S13. Week 7 wild-type maxillary incisor longitudinal section. ***A-B:*** Images taken at 500x magnification of the basal and incisal ends of the incisor from the two sectioning blocks. ***C-L:*** Images taken at 2500x magnification. Arrowheads mark the beginning and end of the post-secretory transition. ***Key:*** Am, ameloblasts; d, dentin; e, enamel; p, pulp.

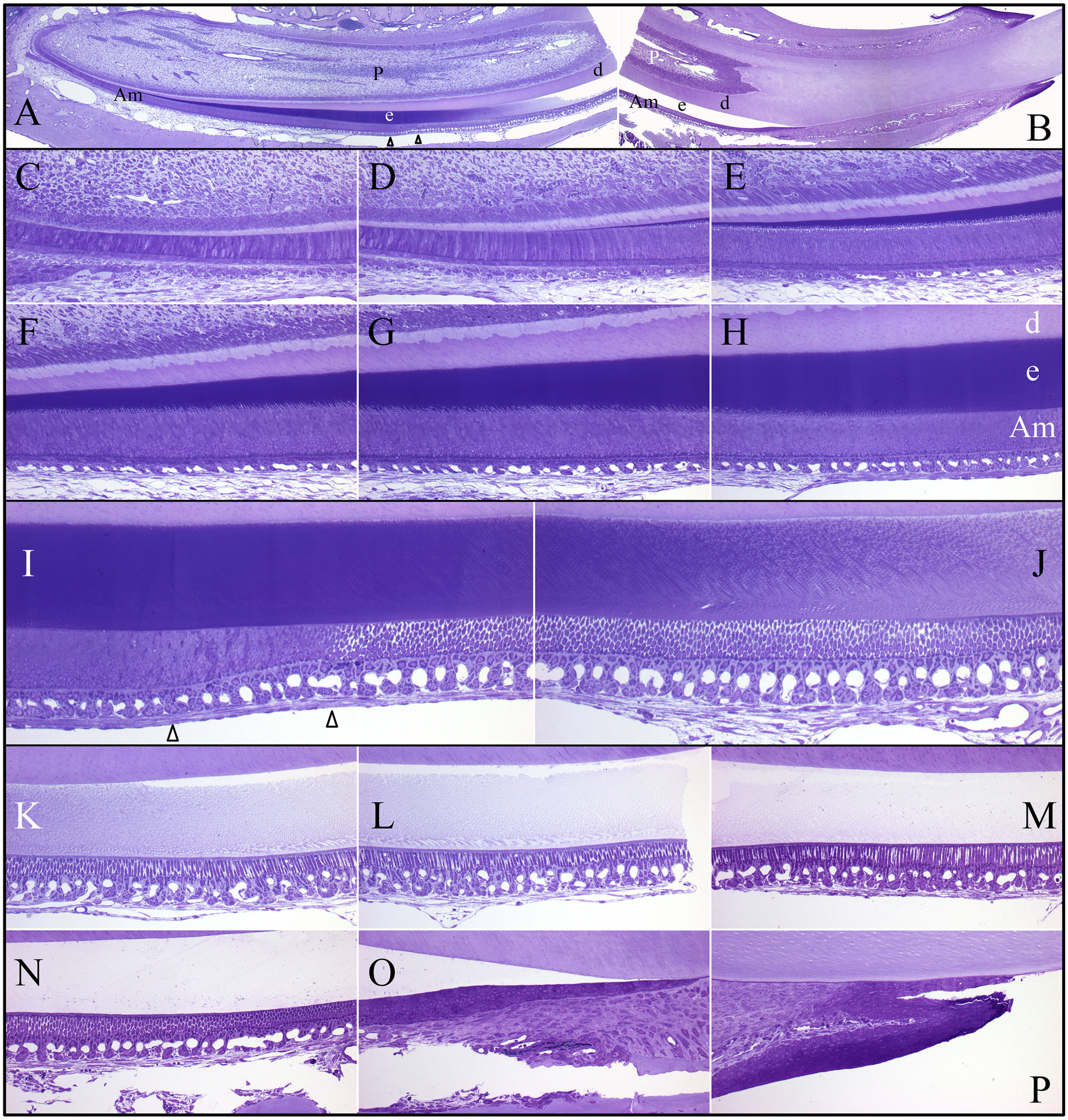

Figure S14. Week 7 wild-type mandibular incisor longitudinal section. ***A-B:*** Images taken at 500x magnification of the basal and incisal ends of the incisor from the two sectioning blocks. ***C-P:*** Images taken at 2500x magnification. Arrowheads mark the beginning and end of the post-secretory transition. ***Key:*** Am, ameloblasts; d, dentin; e, enamel; p, pulp.

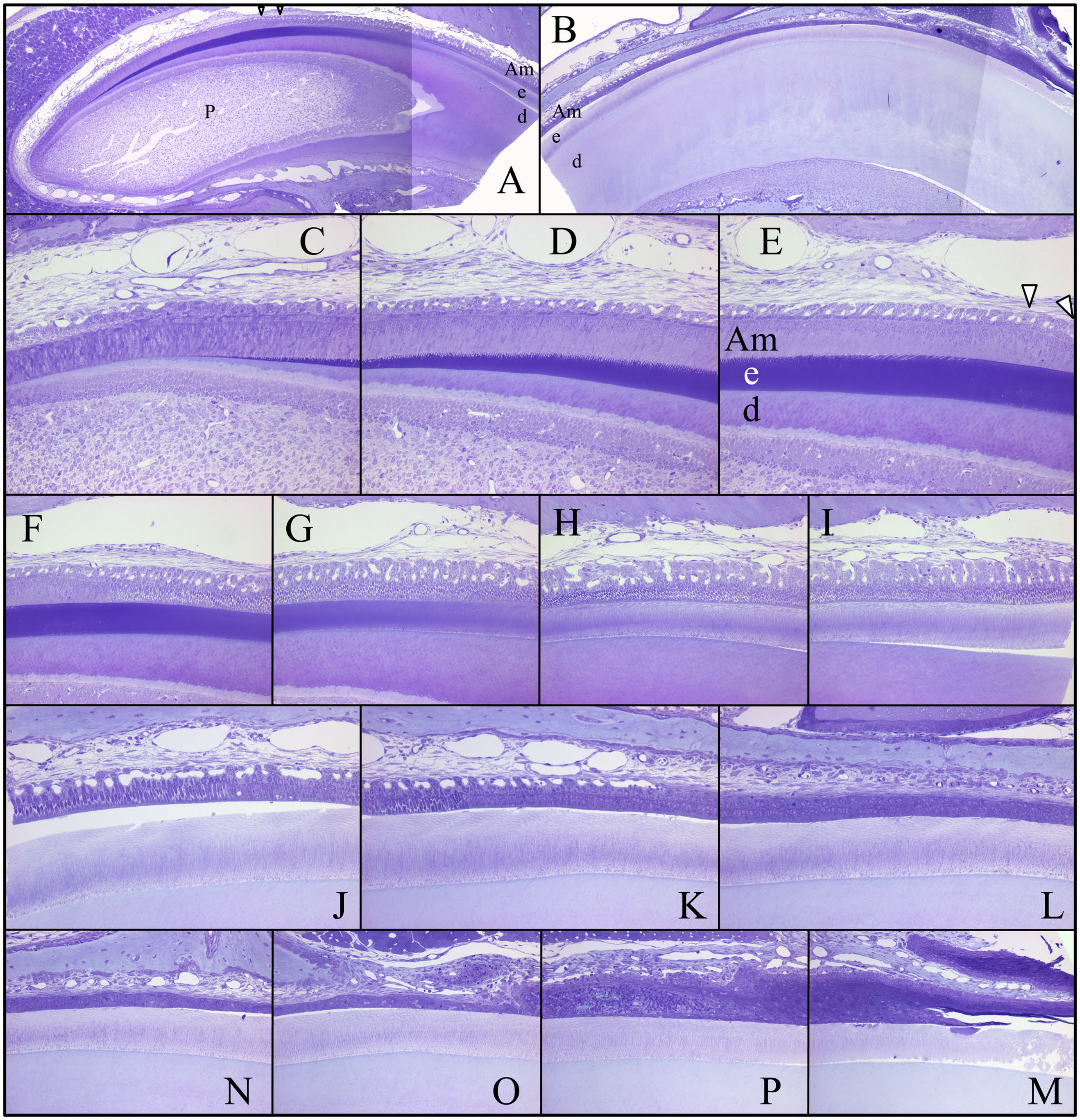

Figure S15. Week 7 *Klk4* null maxillary incisor longitudinal section. ***A-B:*** Images taken at 500x magnification of the basal and incisal ends of the incisor from the two sectioning blocks. ***C-L:*** Images taken at 2500x magnification. Arrowheads mark the beginning and end of the post-secretory transition. ***Key:*** Am, ameloblasts; d, dentin; e, enamel; p, pulp.

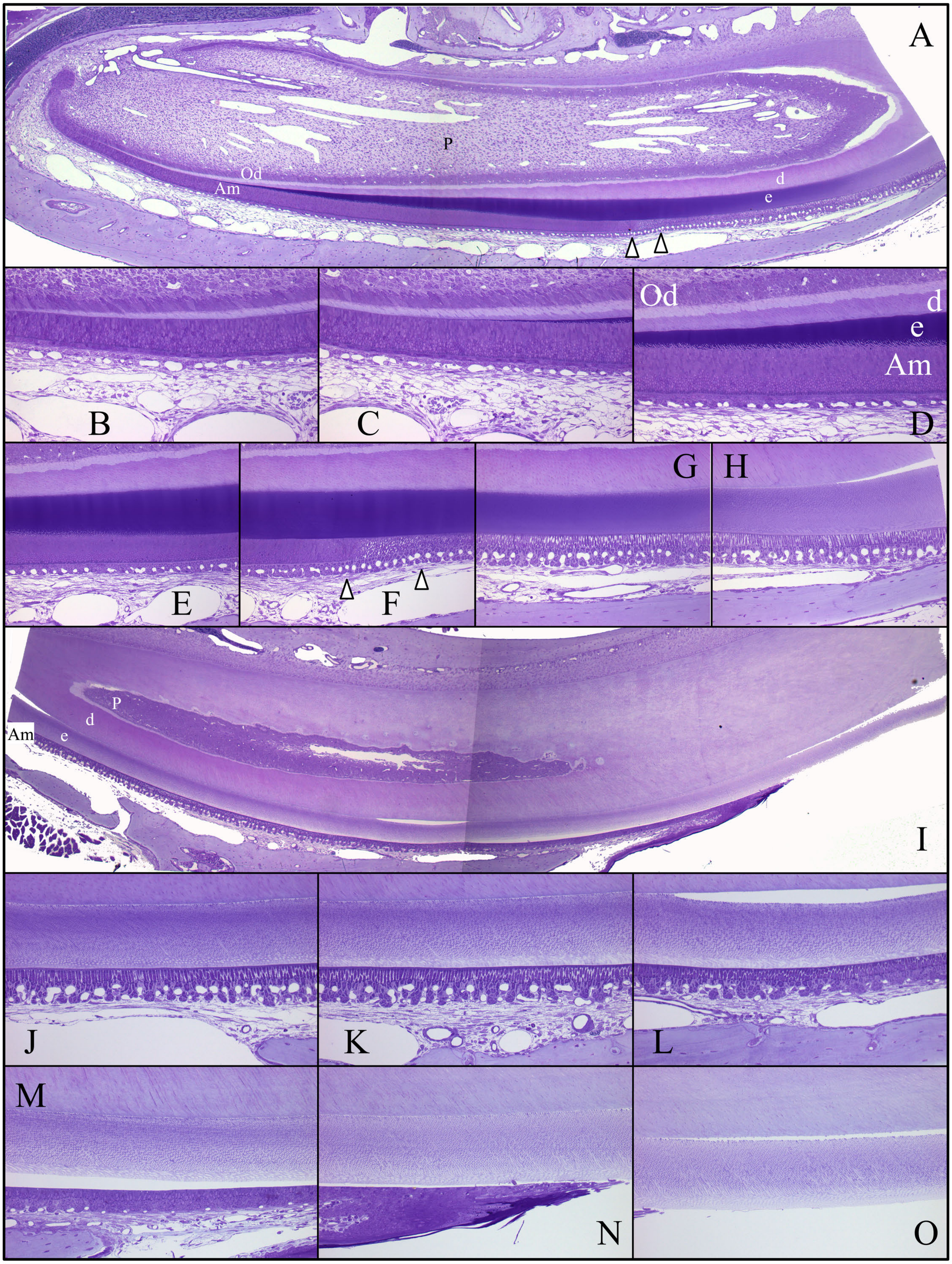

Figure S16. Week 7 *Klk4* null mandibular incisor longitudinal section. ***A and I:*** Images taken at 500x magnification of the basal and incisal ends of the incisor from the two sectioning blocks. ***B-H and J-O:*** Images taken at 2500x magnification. Arrowheads mark the beginning and end of the post-secretory transition. ***Key:*** Am, ameloblasts; d, dentin; e, enamel; p, pulp.

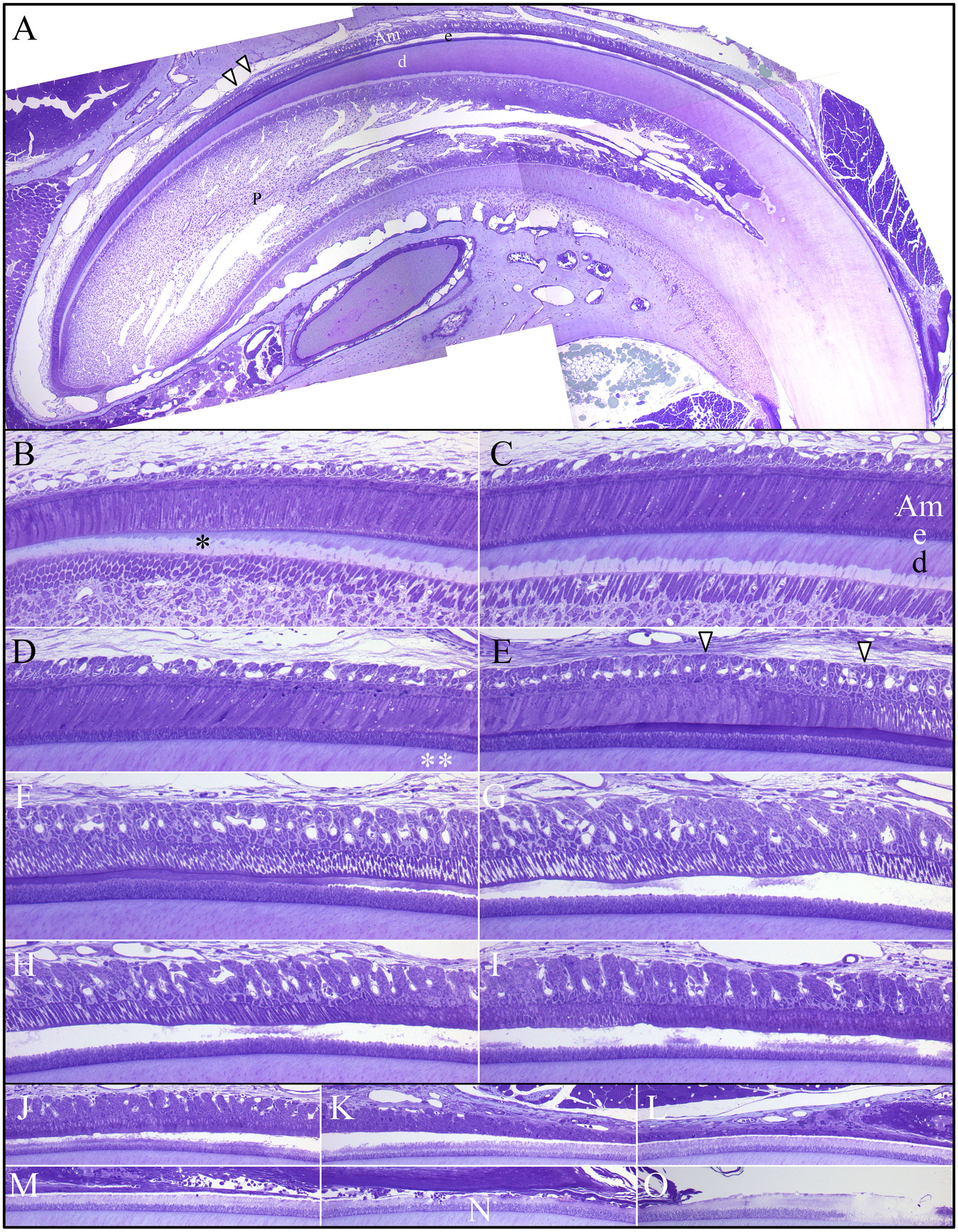

Figure S17. Week 7 *Mmp20* null maxillary incisor longitudinal section. ***A:*** Collage of incisor from images taken at 500x magnification. ***B-O:*** Images taken at 2500x magnification. The single asterisk marks the onset of the secretory stage. The double asterisk marks the onset of a dark stain on the surface of enamel associated with the third (most superficial) mineral layer. Arrowheads mark the beginning and end of the post-secretory transition. ***Key:*** Am, ameloblasts; d, dentin; e, enamel; p, pulp.

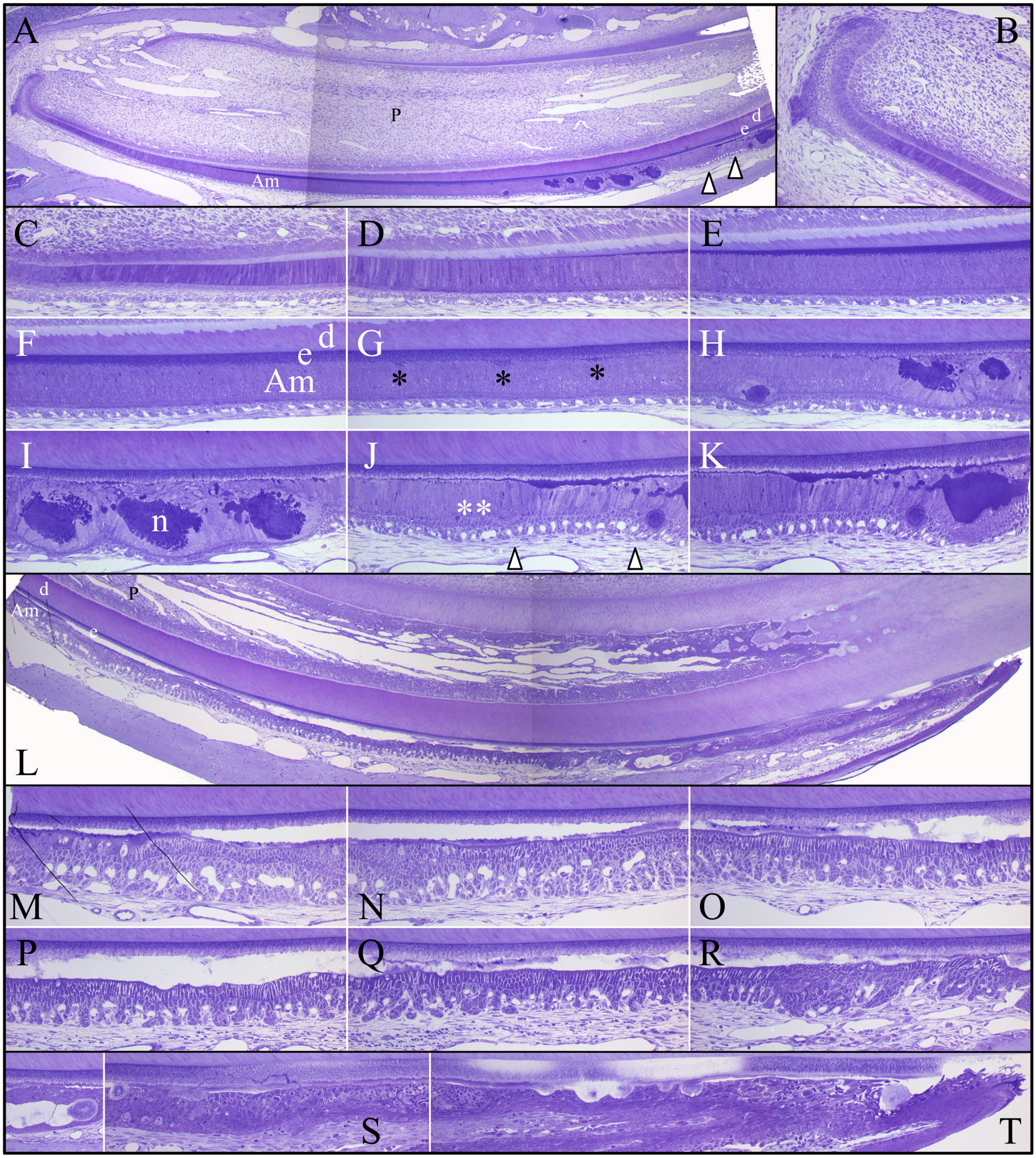

Figure S18. Week 7 *Mmp20* null mandibular incisor longitudinal section. ***A and L:*** Images taken at 500x magnification of the basal and incisal ends of the incisor from the two sectioning blocks. ***B-K and M-T:*** Images taken at 2500x magnification. Arrowheads mark the beginning and end of the post-secretory transition. Dark asterisks mark initial matrix buildup observed in the cellular layer. ***Key:*** Am, ameloblasts; d, dentin; e, enamel; n, nodule; p, pulp.

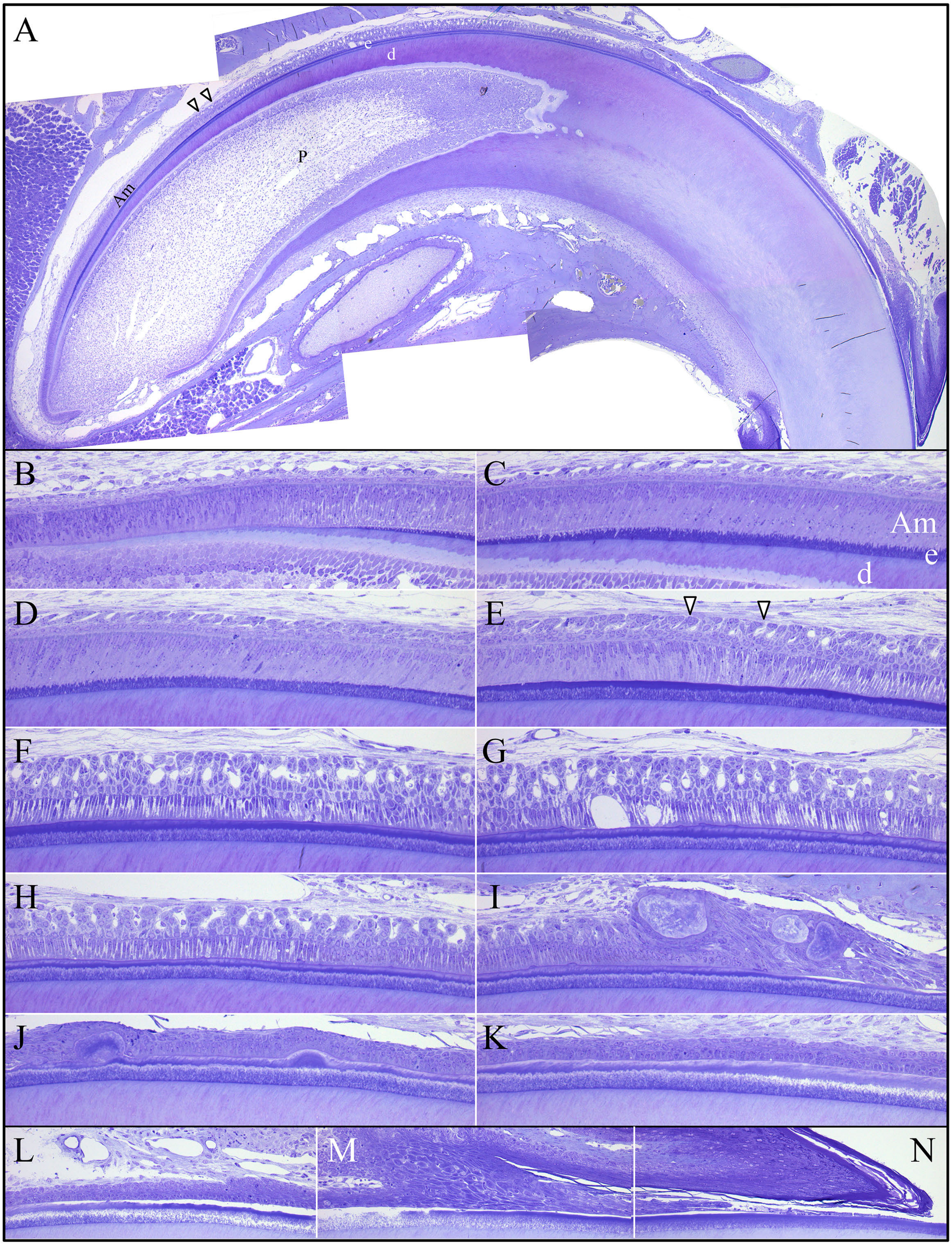

Figure S19. Week 7 *Mmp20*$^{-/-}$*Klk4*$^{-/-}$ maxillary incisor longitudinal section. ***A:*** Collage of incisor from images taken at 500x magnification. ***B-N:*** Images taken at 2500x magnification. Arrowheads mark the beginning and end of the post-secretory transition. ***Key:*** Am, ameloblasts; d, dentin; e, enamel; p, pulp.

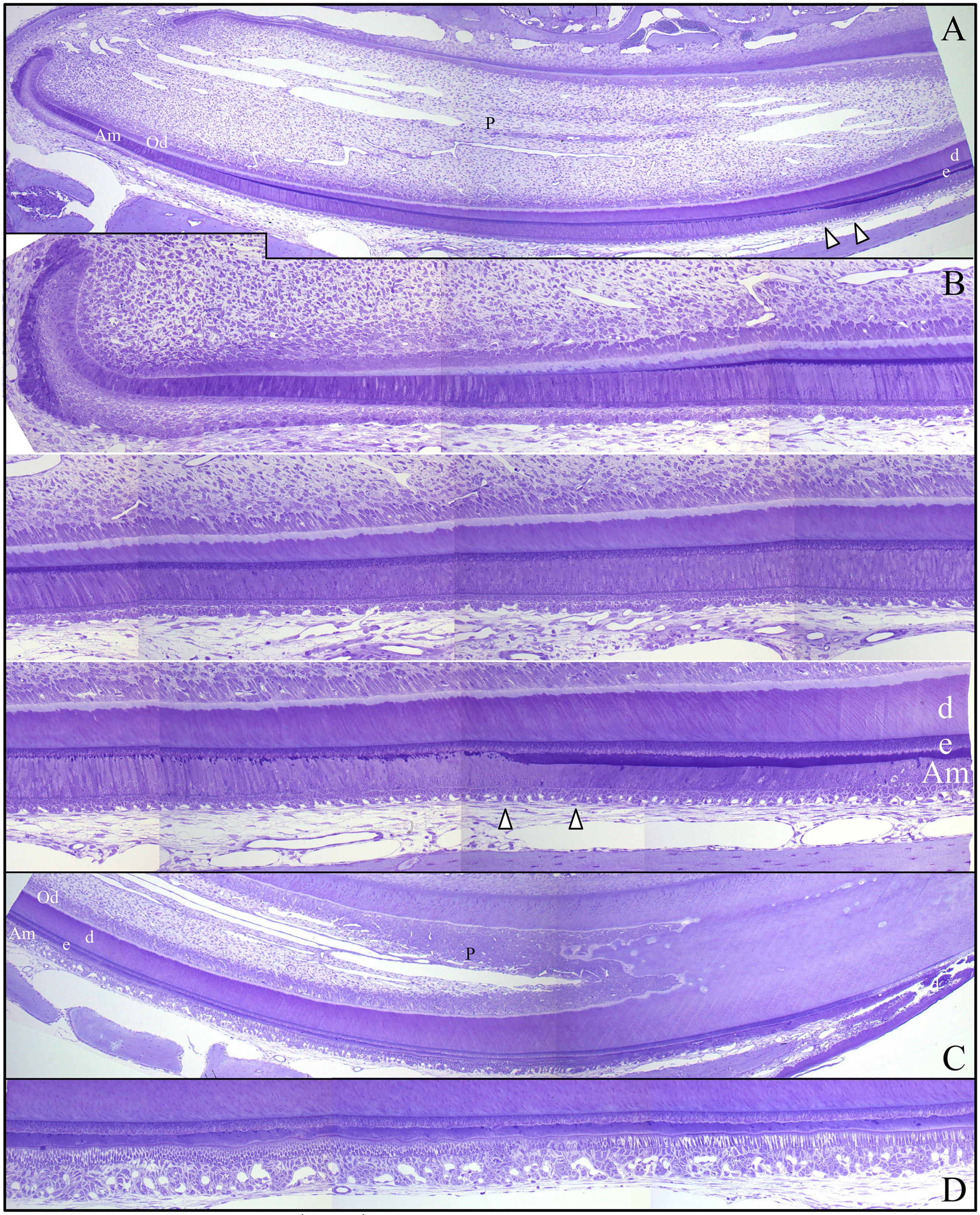

Figure S20. Week 7 *Mmp20*$^{-/-}$*Klk4*$^{-/-}$ mandibular incisor longitudinal section. ***A:*** Images taken at 500x magnification of the basal end of the incisor. ***B:*** Images of the basal end taken at 2500x magnification. Arrowheads mark the beginning and end of the post-secretory transition. ***C:*** Images taken at 500x magnification of the incisal end of the incisor. ***D:*** Images taken at 2500x magnification of the incisal end. ***Key:*** Am, ameloblasts; d, dentin; e, enamel; p, pulp.

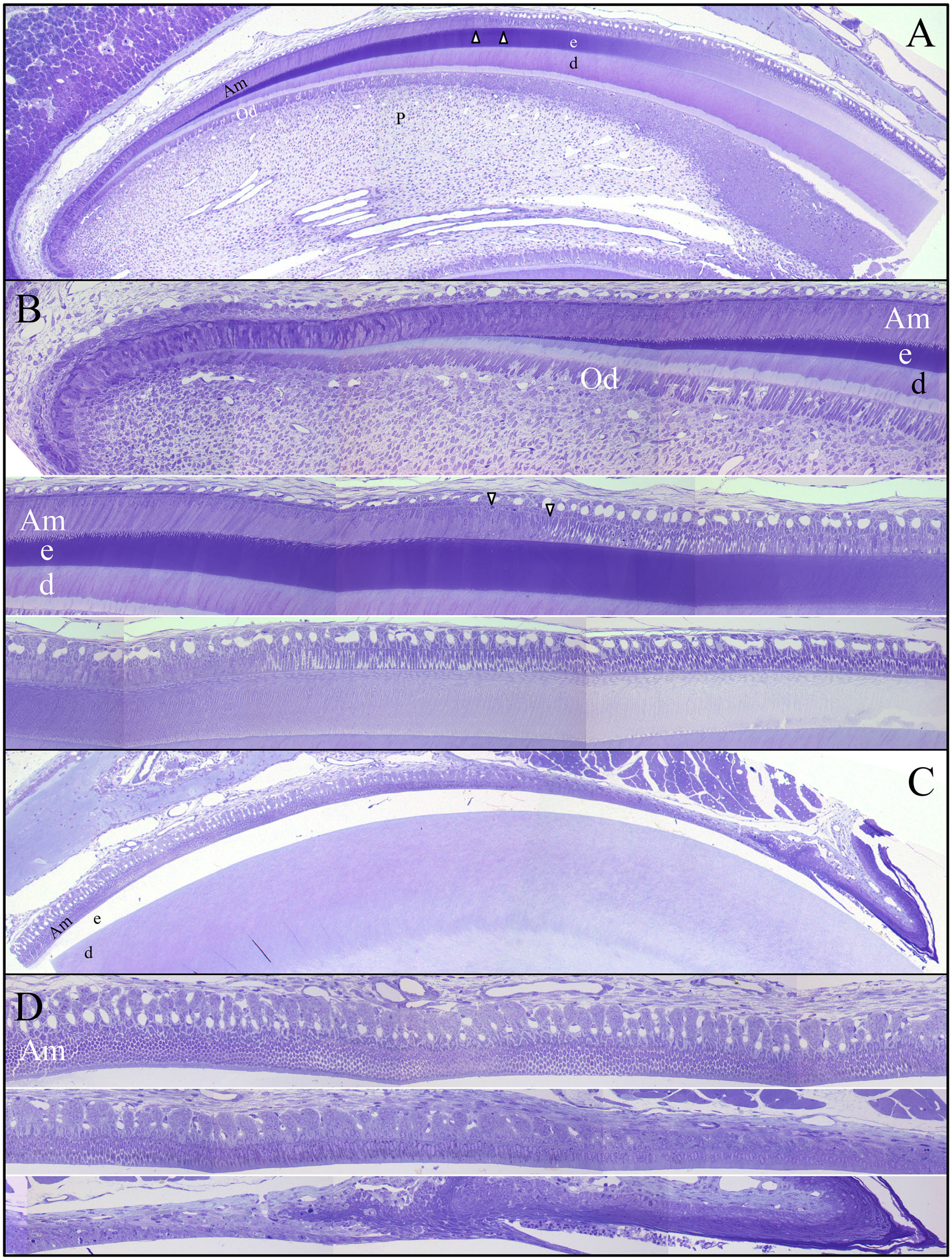

Figure S21. Week 7 *Klk4* heterozygous maxillary incisor longitudinal section. ***A:*** Images taken at 500x magnification of the basal end of the incisor. ***B:*** Images of the basal end taken at 2500x magnification. Arrowheads mark the beginning and end of the post-secretory transition. ***C:*** Images taken at 500x magnification of the incisal end of the incisor. ***D:*** Images taken at 2500x magnification of the incisal end. ***Key:*** Am, ameloblasts; d, dentin; e, enamel; Od, odontoblasts; p, pulp.

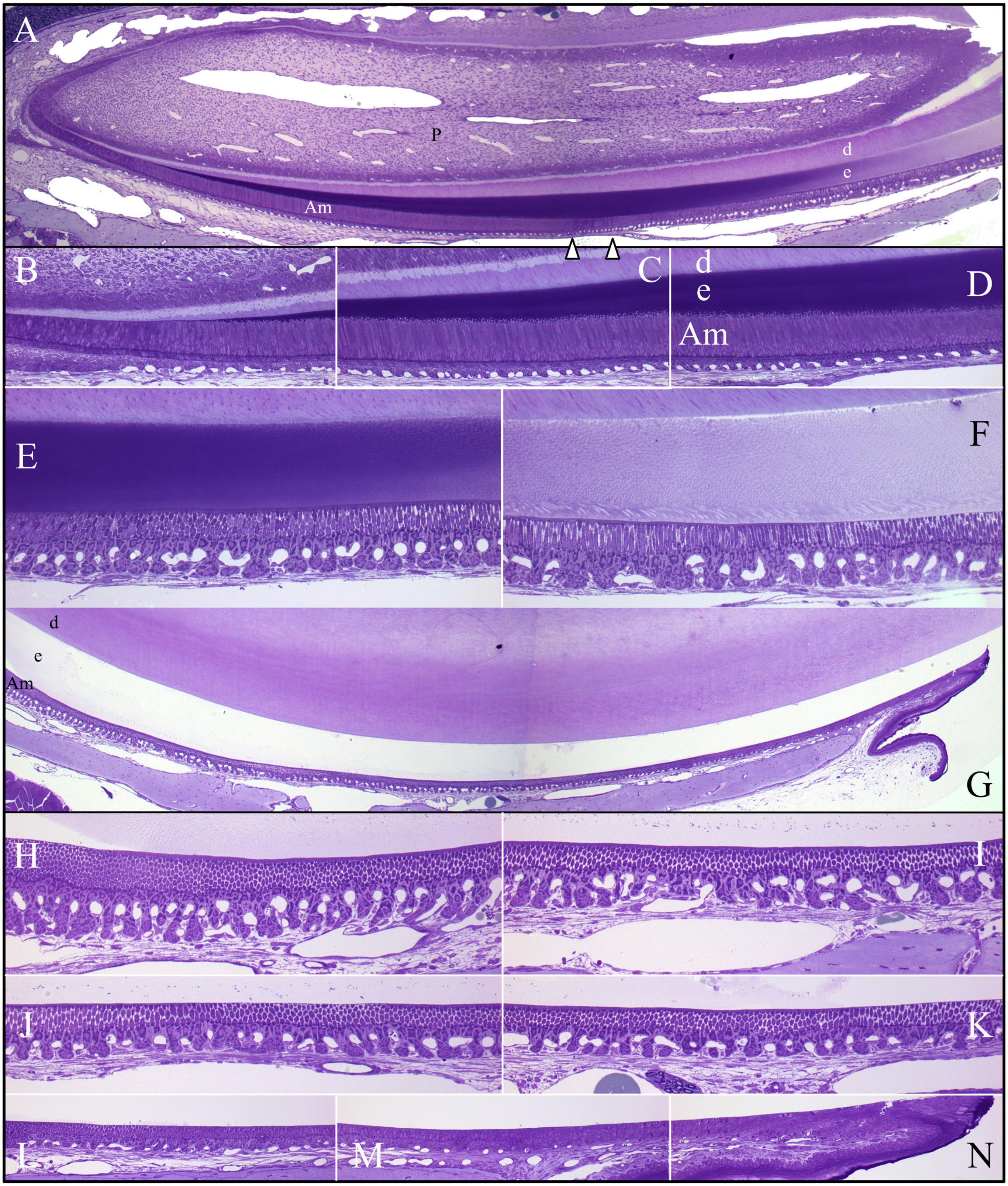

Figure S22. Week 7 *Klk4* heterozygous mandibular incisor longitudinal section. ***A:*** Images taken at 500x magnification of the basal end of the incisor. Arrowheads mark the beginning and end of the post-secretory transition. ***B-F:*** Images of the basal end taken at 2500x magnification. ***G:*** Images taken at 500x magnification of the incisal end of the incisor. ***H-N:*** Images taken at 2500x magnification of the incisal end. ***Key:*** Am, ameloblasts; d, dentin; e, enamel; Od, odontoblasts; p, pulp.

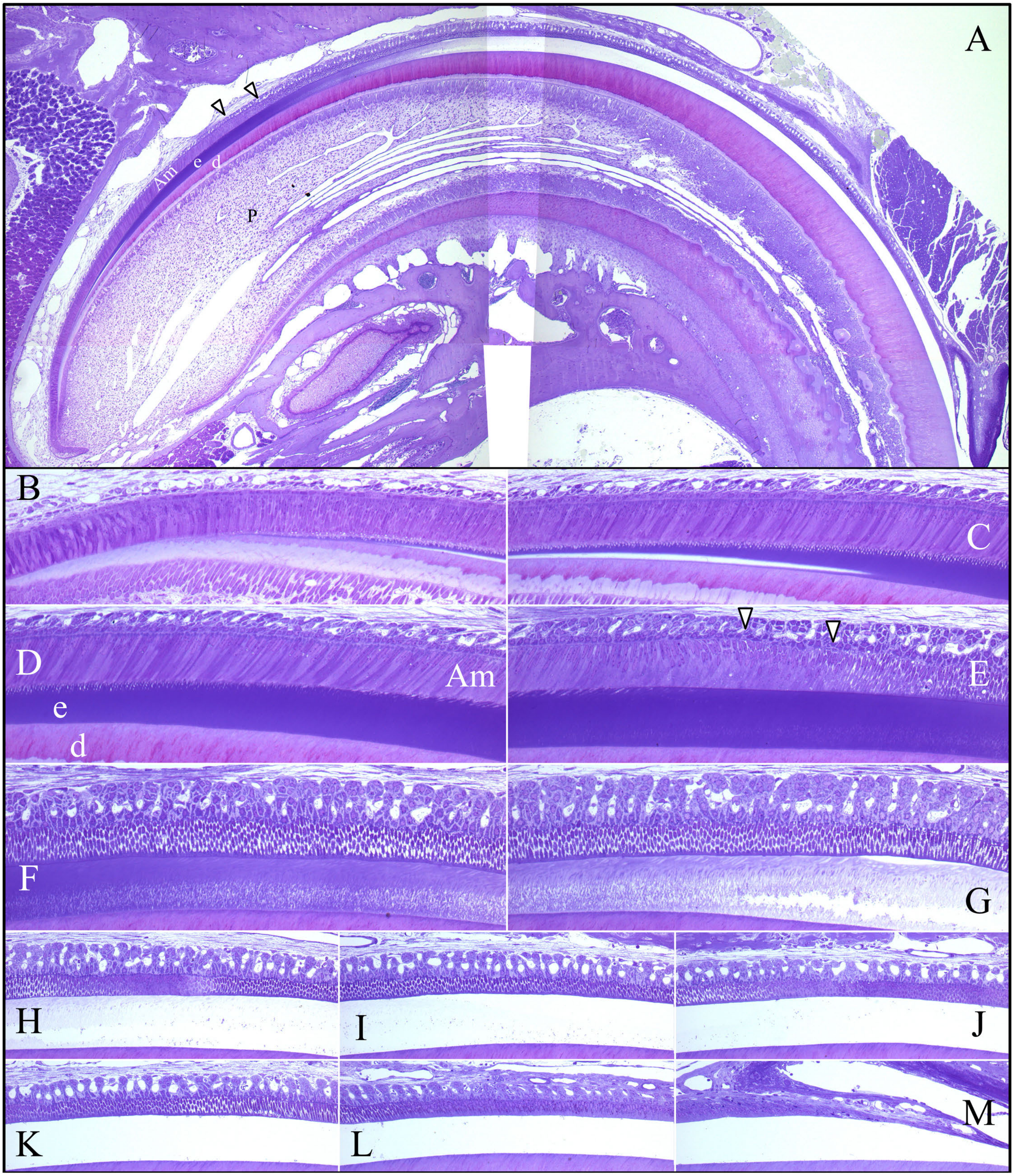

Figure S23. Week 7 *Mmp20* heterozygous maxillary incisor longitudinal section. ***A:*** Collage of incisor from images taken at 500x magnification. ***B-M:*** Images taken at 2500x magnification. Arrowheads mark the beginning and end of the post-secretory transition. ***Key:*** Am, ameloblasts; d, dentin; e, enamel; p, pulp.

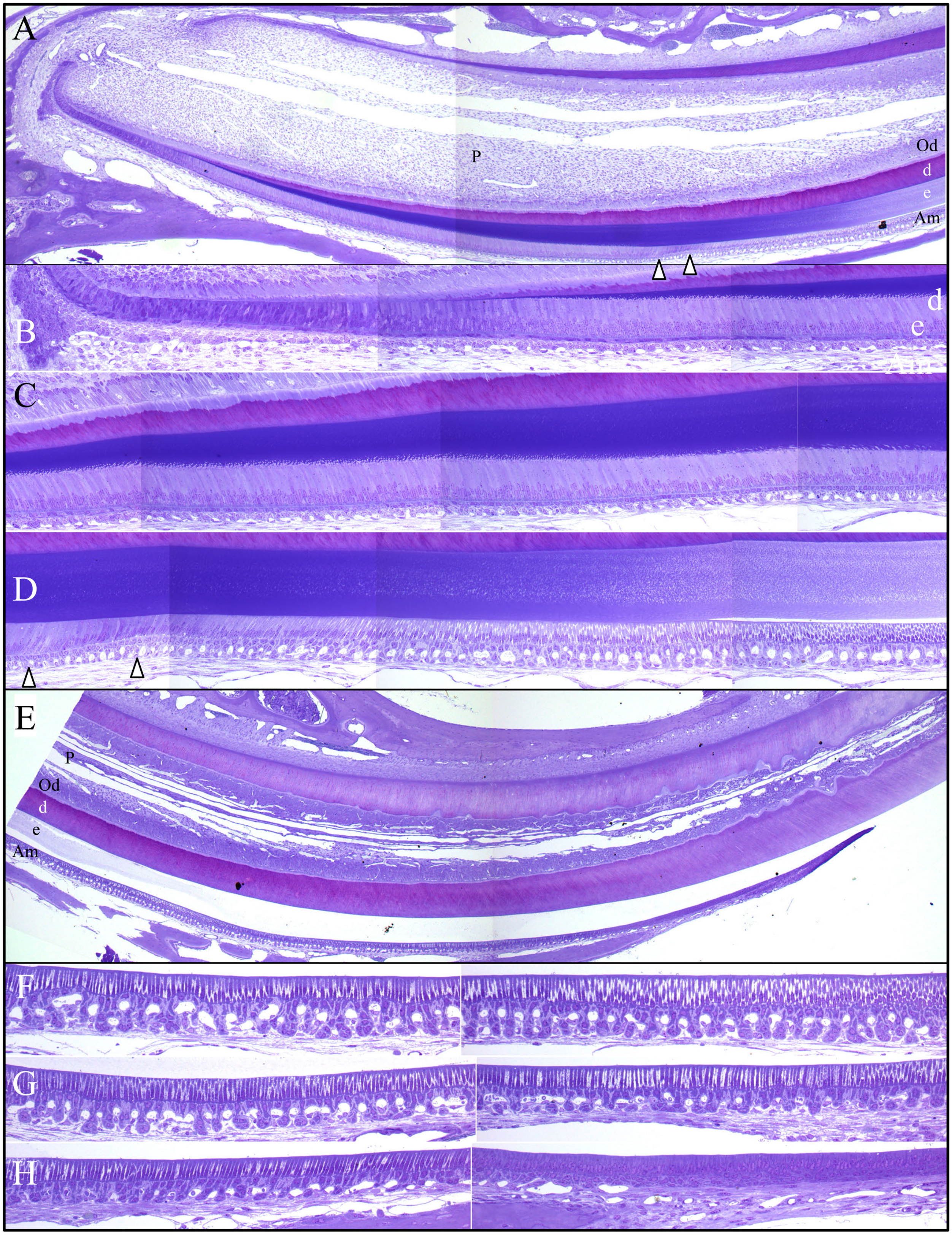

Figure S24. Week 7 *Mmp20* heterozygous mandibular incisor longitudinal section. ***A:*** Images taken at 500x magnification of the basal end of the incisor. Arrowheads mark the beginning and end of the post-secretory transition. ***B-D:*** Images of the basal end taken at 2500x magnification. ***E:*** Images taken at 500x magnification of the incisal end of the incisor. ***F-H:*** Images taken at 2500x magnification of the incisal end. ***Key:*** Am, ameloblasts; d, dentin; e, enamel; Od, odontoblasts; p, pulp.

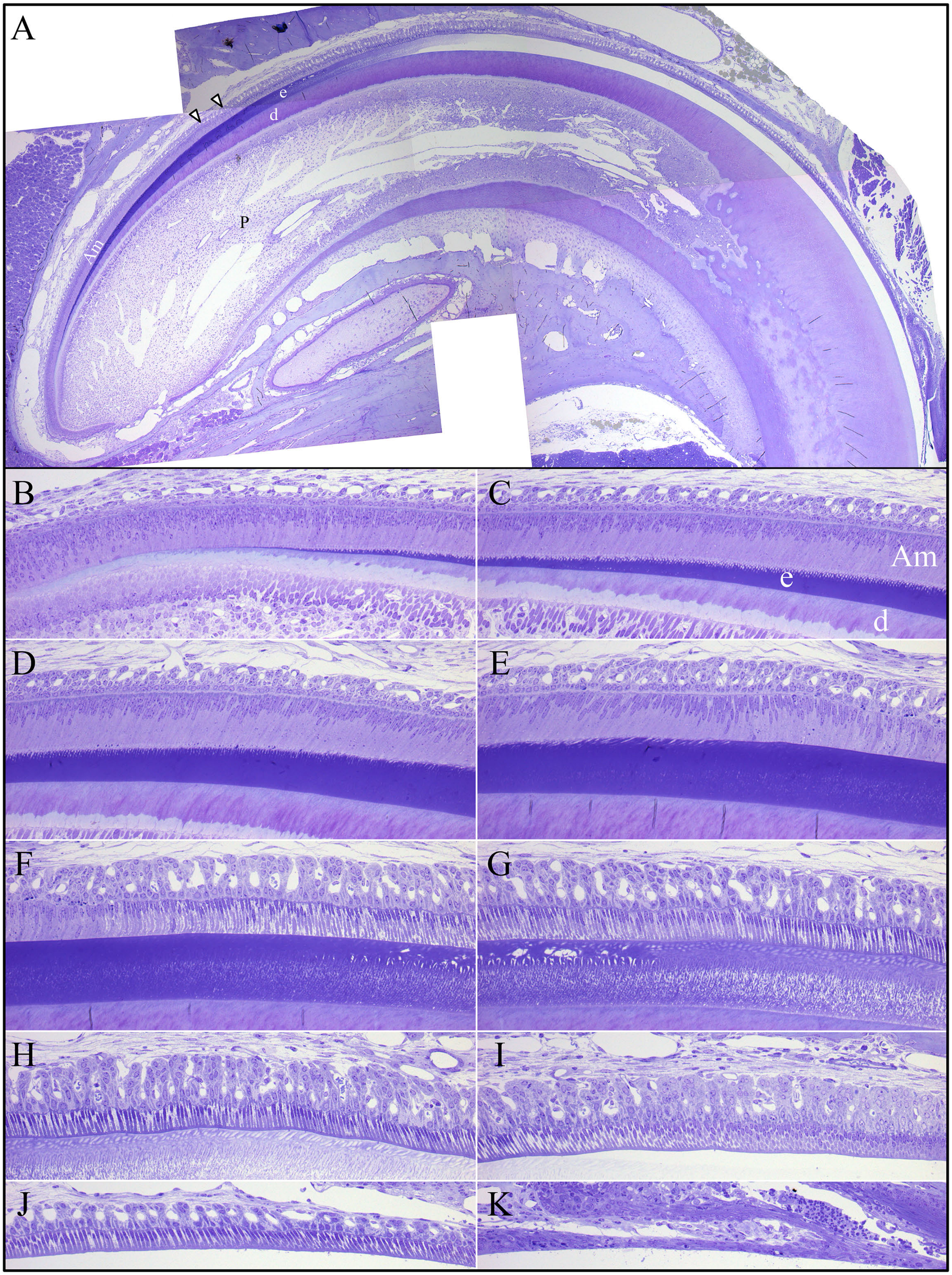

Figure S25. Week 7 *Mmp20*/Klk4 double heterozygous maxillary incisor longitudinal section. ***A:*** Collage of incisor from images taken at 500x magnification. ***B-K:*** Images taken at 2500x magnification. Arrowheads mark the beginning and end of the post-secretory transition. ***Key:*** Am, ameloblasts; d, dentin; e, enamel; p, pulp.

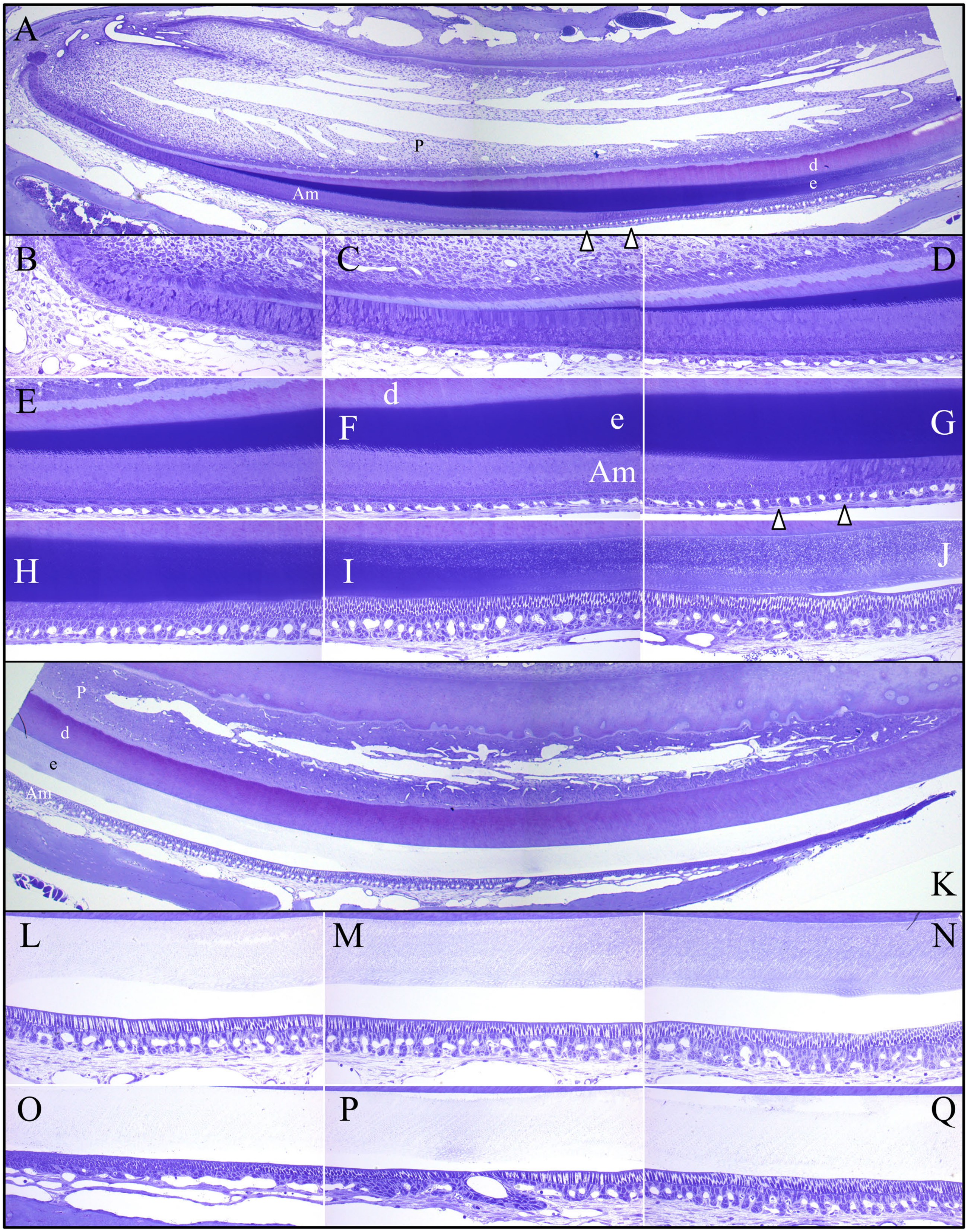

Figure S26. Week 7 *Mmp20*/Klk4 double heterozygous mandibular incisor longitudinal section. ***A:*** Images of the basal end of the incisor (taken at 500x). ***B-J:*** Images of the basal end (taken at 2500x). ***K:*** Images taken at 500x magnification of the incisal end of the incisor. ***L-Q:*** Images taken at 2500x magnification of the incisal end. ***Key:*** Am, ameloblasts; d, dentin; e, enamel; Od, odontoblasts; p, pulp. Arrowheads mark the beginning and end of the post-secretory transition.
